# Genomic landscape and its correlations with tumor mutational burden, PD-L1 expression, and immune cells infiltration in Chinese lung squamous cell carcinoma

**DOI:** 10.1186/s13045-019-0762-1

**Published:** 2019-07-12

**Authors:** Tao Jiang, Jinpeng Shi, Zhengwei Dong, Likun Hou, Chao Zhao, Xuefei Li, Beibei Mao, Wei Zhu, Xianchao Guo, Henghui Zhang, Ji He, Xiaoxia Chen, Chunxia Su, Shengxiang Ren, Chunyan Wu, Caicun Zhou

**Affiliations:** 1 Department of Medical Oncology, Shanghai Pulmonary Hospital & Thoracic Cancer Institute, Tongji University School of Medicine, Zheng Min Road, Shanghai, 200433 China; 2 Department of Pathology, Shanghai Pulmonary Hospital & Thoracic Cancer Institute, Tongji University School of Medicine, Shanghai, 200433 China; 3 Department of Lung Cancer and Immunology, Shanghai Pulmonary Hospital, Tongji University School of Medicine, No. 507, Zheng Min Road, Shanghai, 200433 China; 4 Beijing Genecast Biotechnology Co., Beijing, 100191 China

**Keywords:** Lung squamous cell carcinoma, Genome, TMB, PD-L1, Immune cells

## Abstract

**Introduction:**

To depict the genomic landscape of Chinese early-stage lung squamous cell carcinoma (LUSC) and investigate its correlation with tumor mutation burden (TMB), PD-L1 expression, and immune infiltrates.

**Methods:**

Whole-exome sequencing was performed on 189 surgically resected LUSC. TMB was defined as the sum of nonsynonymous single nucleotide and indel variants. CD8^+^ tumor-infiltrating lymphocyte (TIL) density and PD-L1 expression were evaluated by immunohistochemistry. Six immune infiltrates were estimated using an online database.

**Results:**

The median TMB was 9.43 mutations per megabase. Positive PD-L1 expression and CD8^+^ TILs density were identified in 24.3% and 78.8%. *PIK3CA* amplification was associated with significantly higher TMB (*P* = 0.036). Frequent genetic alterations had no impact on PD-L1 expression but *PIK3CA* amplification and *KEAP1* mutation were independently associated with significantly lower CD8^+^ TIL density (*P* < 0.001, *P* = 0.005, respectively). Low TMB and high CD8^+^ TIL density were independently associated with longer disease-free survival (DFS) while none of them could individually predict the overall survival (OS). Combination of TMB and PD-L1 expression or TMB and CD8^+^ TIL density could stratify total populations into two groups with distinct prognosis. Classifying tumor-immune microenvironment based on PD-L1 expression and CD8^+^ TIL density showed discrepant genomic alterations but similar TMB, clinical features, and OS. Notably, patients with different smoking status had distinct prognostic factors.

**Conclusion:**

The combination of TMB, PD-L1 expression, immune infiltrates, and smoking status showed the feasibility to subgroup stratification in Chinese patients with early-stage LUSC, which might be helpful for future design of personalized immunotherapy trials in LUSC.

**Electronic supplementary material:**

The online version of this article (10.1186/s13045-019-0762-1) contains supplementary material, which is available to authorized users.

## Introduction

Lung squamous cell carcinoma (LUSC) is one of the most common histological subtypes of non-small-cell lung cancer (NSCLC), the remaining leading cause of cancer-related death worldwide for a long period [1–3]. Unlike lung adenocarcinoma (LUAD) with oncogenic driver alterations, therapeutic progress for LUSC is limited and conventional platinum-based chemotherapy remains the standard-of-care for many years [4–6]. Recently, immune checkpoint inhibitors targeted programmed cell death 1 (PD-1) and its ligand (PD-L1) has a shift the paradigm in both LUAD and LUSC. To date, several anti-PD-1/PD-L1 antibodies including nivolumab, pembrolizumab, and atezolizumab have been approved as second-line settings for patients with advanced NSCLC [7–11]. Moreover, pembrolizumab monotherapy showed superiority as a first-line setting when compared with chemotherapy in patients with PD-L1 tumor proportion score (TPS) > 1%, and pembrolizumab plus platinum-based chemotherapy become the standard-of-care for first-line setting patients with LUSC [11, 12]. Emerging evidence indicated that positive PD-L1 expression and high tumor mutation burden (TMB) could predict the response to anti-PD-1/PD-L1 therapies in NSCLC [12, 13]. However, the ideal predictive biomarkers for immunotherapy are still an unmet need in clinical practice. Inspiringly, latest reports found gene profiling showed the potent to predict response to immune checkpoint inhibitors [14–16].

For patients with early-stage LUSC, curative surgery with adjuvant chemotherapy is the main therapeutic option even though adjuvant chemotherapy just brought limited survival benefit [17]. Recently, neoadjuvant and adjuvant immune checkpoint inhibitors showed promising results in this setting. A pilot study found that neoadjuvant nivolumab resulted in a major pathological response of 45% [18]. Further biomarker analysis found that TMB was predictive of the pathological response to neoadjuvant nivolumab, while in LCMC3 study, TMB did not associate with pathological response for neoadjuvant atezolizumab and response was found in patients with PD-L1 expression negative [19]. Therefore, comprehensively depicting the genomic and immune landscape, their correlations are needed to elucidate the ideal phenotype to benefit neo-adjuvant immunotherapy in patients with early-stage NSCLC. Although several studies have reported the relevant data on LUAD [20, 21], the situation in LUSC is still largely unknown.

Herein, we performed this study in 189 Chinese patients with early-stage LUSC to evaluate (1) the genomic landscape of LUSC and its correlation with PD-L1 expression, TMB, and six immune infiltrates and (2) their associations with clinical parameters, disease-free survival (DFS), and overall survival (OS). Additionally, we also compared our data to other ethnicities and tumor types such as adenocarcinoma.

## Methods

### Sample collection

We retrospectively identified patients who underwent surgical resection of the lung (lobectomy or pulmonectomy) and histologically confirmed LUSC at Shanghai Pulmonary Hospital from 2012 to 2015. We firstly checked the histological types of each case using medical electronic records. Then all primary diagnoses were further independently evaluated by two experienced pathologists (Z.W.D and L.K.H) according to the WHO nomenclature for squamous carcinoma. The specimens of eligible case must have a confirmed diagnosis of LUSC and had at least 50% tumor cellularity. Major exclusion criteria were inadequate or poor quality samples, missing baseline clinicopathological features, mixed histology, and incomplete follow-up data. Corresponding formalin-fixed and paraffin-embedded (FFPE) tissues were used for immunohistochemistry (IHC) staining and whole-exome sequencing. The major baseline features including age, sex, smoking history, Eastern Cooperative Oncology Group performance status (ECOG PS), tumor size, node status and stage, vascular invasion, differentiation degree, tumor stage, DFS, and OS. A never-smoker was defined as a patient who had smoked less than 100 cigarettes during his/her lifetime. DFS was defined as the time from the initial surgical resection until recurrence. OS was calculated from the date of LUSC diagnosis to death from any cause or was censored at the last follow-up date. This study was conducted in accordance with the provisions of the Declaration of Helsinki and was approved by the ethics committee of Shanghai Pulmonary Hospital.

### PD-L1 expression

PD-L1 expression was firstly tested by using anti-human PD-L1 (#13684, clone E1L3N, Cell Signaling Technology, Danvers, MA, diluted 1:200) according to the manufacturer’s recommendations and previous publications using 4–5 μm FFPE sections [22, 23]. Then all of the samples were re-evaluated by using another antibody assay (clone SP142, Spring Bioscience, Ventana, Tucson, AZ, diluted 1:100) [24]. For E1L3N staining, PD-L1 expression was defined as the percentage of tumor cells showing membranous immunoreactivity (central or marginal tumor region). The cutoff value was 5% for PD-L1 positivity or negativity (PD-L1^+/−^). PD-L1 > 50% was defined as PD-L1 strong positivity. For SP142 staining, positive cells were defined as cancer cells displaying membranous staining for PD-L1, and the proportion of PD-L1^+^ cells was evaluated as the percentage of total cancer cells in whole sections. The cutoff values of 1%, 5%, 10%, and 50% were set and 1% was determined for PD-L1^+/−^ [25, 26]. Sections from human placenta tissues were used as the positive controls of PD-L1 IHC staining. Breast cancer cell line (MCF-7) was utilized as the negative control. All of the stained sections were independently reviewed by two pathologists (Z.W.D and L.K.H). Any discrepancies were discussed together and a consensus was achieved under the guidance of another experienced pathologist (C.Y.W).

### CD8^+^ tumor-infiltrating lymphocyte (TIL) density

CD8^+^ TILs density was assessed by using a mouse anti-CD8 monoclonal antibody (M7103, clone C8144B, DAKO). Lymphocytes with immunostained CD8 infiltrating within a tumor region (central or marginal) were defined as CD8^+^ TILs. On the basis of the percentage of CD8^+^ TILs displayed within a tumor region, we determined high/low CD8^+^ TIL density (CD8^+^ TIL^+/−^) with a cutoff of 5%, which was analogous to the previous studies [23, 27].

### TMB calculation

The details of whole-exome sequencing and data processing were listed in Additional file 1. TMB was defined as the number of somatic, coding, base substitution, and indel mutations per megabase of genome examined by using nonsynonymous and frameshift indels at 5% limit of detection. Variants in low confidence regions and repeat regions, driver mutations and germline mutations were removed via using population online databases (The Exome Aggregation Consortium v.03, Genome Aggregation Database, and 1000 Genome) followed by variant allelic frequency cutoff of 0.2%. The tumor mutation calculation formula was as follows:

$$ \mathrm{TMB}=\frac{S_n\times 1000000}{N} $$

*S*_*n*_ represents the absolute number of somatic mutations, and *N* represents the number of exonic bases coverage depth ≥ 100 ×.

### Estimation of immune cells infiltration

The abundances of six immune cell infiltrations including B cells, CD4^+^ T cells, CD8^+^ T cells, macrophages, neutrophils, and dendritic cells (DC) in specific groups of LUSC were estimated by using online database, named Tumor Immune Estimation Resource (TIMER). TIMER is a comprehensive resource for systematical analysis of immune cell infiltrations across diverse cancer types, which is validated using pathological estimations. The details and statistical methods were listed in this website (https://cistrome.shinyapps.io/timer/) and their previous publications [28, 29].

### Statistical analysis

The Pearson correlation coefficient was used to determine the correlation of PD-L1 expression level between two antibody assays. Spearman’s rank correlation was utilized to assess the correlations among PD-L1 expression, TMB, and CD8^+^ TIL density. Correlations between PD-L1 expression/CD8^+^ TIL density and clinical parameters were analyzed using the chi-squared or Fisher’s exact test for categorical variables. The continuous variable was analyzed by ANOVA and Tukey’s multiple comparison tests. Mann-Whitney *U* tests or Kruskal-Wallis rank sum tests were used for comparisons of continuous variables across multiple groups. The Kaplan-Meier curve was leveraged to assess the patients’ survival curves. The log-rank test was used to test the significance of differences between two or four groups. Cox proportional hazards model was utilized for uni- and multivariate survival analyses to calculate the hazard ratios (HR) and related 95% confidence intervals (CI). *P* < 0.05 was considered significant. All statistical analyses were performed using GraphPad PRISM 6.0 and the SPSS statistical software, version 22.0 (SPSS Inc., Chicago, IL, USA).

## Results

### Genomic landscape of Chinese LUSC and its correlations with TMB, PD-L1 expression, or immune cells infiltration

**Fig. 1:**
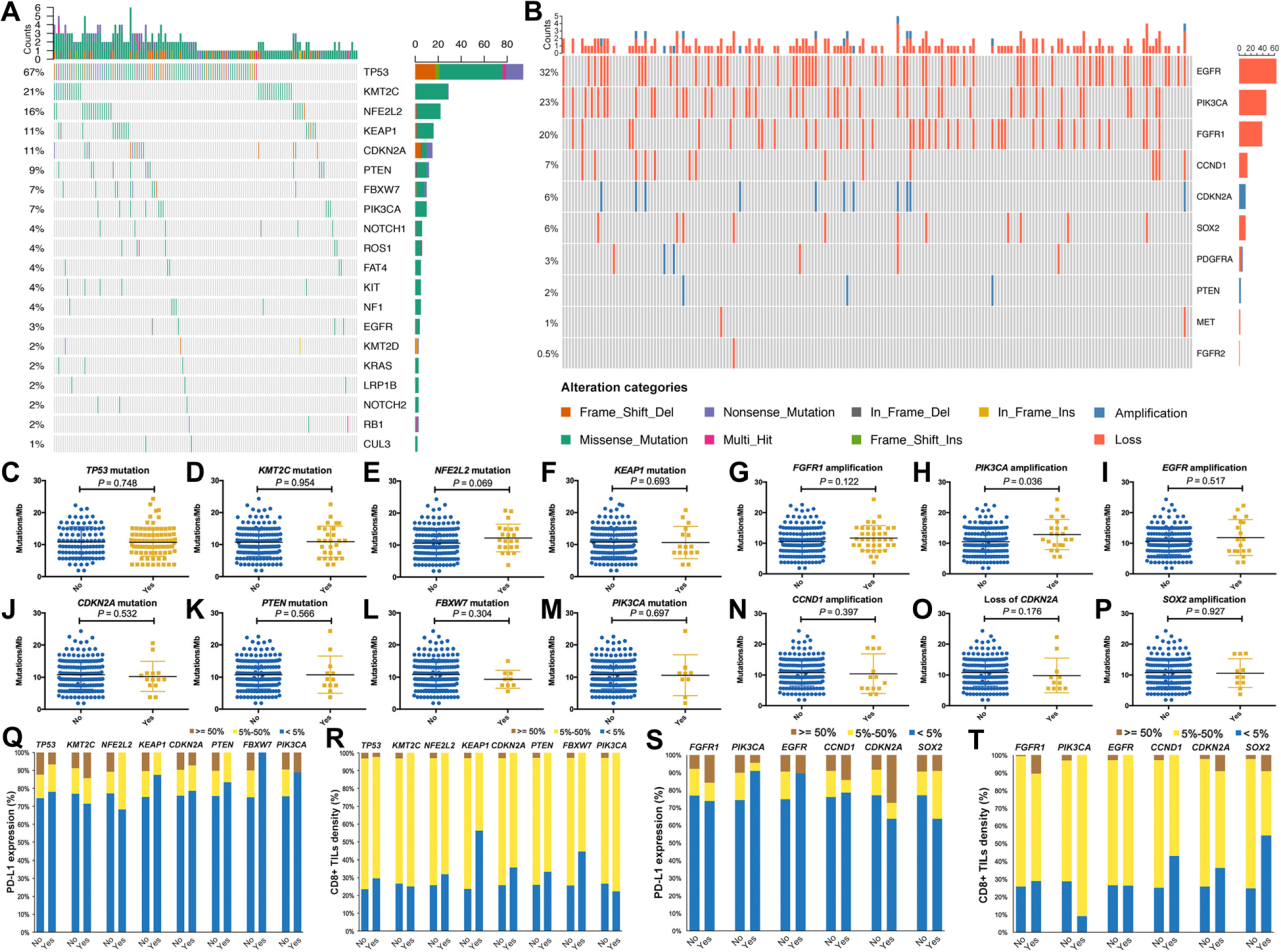
Genomic landscape of Chinese LUSC and its correlations with TMB, PD-L1 expression, or immune cells infiltration. **a** The identified somatic mutations of all cases. **b** The identified copy number variations of all samples. **c**–**p** Association between frequent alterations including *TP53* (**c**), *KMT2C* (**d**), *NFE2L2* (**e**), *KEAP1* (**f**), *CDKN2A* (**j**), *PTEN* (**k**), *FBXW7* (**l**) and *PIK3CA* (**m**) mutations, *FGFR1* (**g**), *PIK3CA* (**h**), *EGFR* (**i**), *CCND1* (**n**) and *SOX2* (**p**) amplification, loss of *CDKN2A* (**o**), and TMB. **q**–**r** Association between frequent somatic mutations and PD-L1 expression (**q**), CD8+ TIL expression (**r**). **s**–**t** Association between frequent copy number variations and PD-L1 expression (**s**), CD8+ TIL expression (**t**)

One hundred eighty-nine samples were successfully sequenced. The baseline characteristics were listed in Table 1. The median age was 63 years (range 36–80 years). One hundred seventy-nine (94.7%) patients were male. The percentage of never-smokers (31.7%) was higher than those in previous studies [30, 31]. Ninety-two (48.7%) patients had pathological stage I disease and most of them had PS 0–1. The mean sequencing coverage across all regions was 101 × with 96.4% of targeted bases above 20 × coverage. We identified eight genes with a somatic mutation frequency > 5% of all cases (Fig. 1a): *TP53*, *KMT2C*, *NFE2L2*, *KEAP1*, *CDKN2A*, *PTEN*, *FBXW7*, and *PIK3CA*. This would have a slight difference to TCGA which identified 10 genes (*TP53*, *CDKN2A*, *PTEN*, *PIK3CA*, *KEAP1*, *MLL2*, *HLA-A*, *NFE2L2*, *NOTCH1*, and *RB1*) with a false discovery rate (FDR) *Q* value < 0.1 [30]. In Caucasian with LUSC (Choi et al. cohort), 14 genes exhibited significant enrichment for somatic mutation: *TP53*, *MLL2*, *PIK3CA*, *NFE2L2*, *CDH8*, *KEAP1*, *PTEN*, *ADCY8*, *PTPRT*, *CALCR*, *GRM8*, *FBXW7*, *RB1*, *and CDKN2A* [19]. *TP53* mutation was found in 67% of all cases (TCGA, 81%; Choi et al. cohort, 60%). There were 10 genes with significant copy number variations (CNVs; Fig. 1b): *EGFR*, *PIK3CA*, *FGFR1*, *CCND1*, *CDKN2A*, *SOX2*, *PDGFRA*, *PTEN*, *MET*, and *FGFR2*. *EGFR* amplification (32%) was the most frequent. *PIK3CA* and *FGFR1* amplifications were found in 23% and 20% of all cases, respectively. Interestingly, we found a high frequency of *EML4-ALK* fusion (3.2%), one of the most common driver gene alterations in LUAD. There was no significant correlation between *EGFR* amplification or *EML4-ALK* fusion and clinicopathological features (Additional file 1: Table S1).

**Table 1:** Baseline characteristics of included patients (*n* = 189)

		Total	PD-L1^−^		PD-L1^+^		*P* value	CD8^+^ TIL high		CD8^+^ TIL low		*P* value	TMB high		TMB low		*P* value
		*N*	*N*	%	*N*	%		*N*	%	*N*	%		*N*	%	*N*	%	
Age	< 65	105	72	68.57	33	31.43	0.011	82	78.10	23	21.90	0.113	53	50.48	52	49.52	0.948
	≥ 65	84	71	84.52	13	15.48		57	67.86	27	32.14		42	50.00	42	50.00	
Sex	Male	179	134	74.86	45	25.14	0.480	131	73.18	48	26.82	0.915	91	50.84	88	49.16	0.732
	Female	10	9	90.00	1	10.00		8	80.00	2	20.00		4	40.00	6	60.00	
Smoking	Never	60	49	81.67	11	18.33	0.190	47	78.33	13	21.67	0.309	25	41.67	35	58.33	0.107
	Current/former	129	94	72.87	35	27.13		92	71.32	37	28.68		70	54.26	59	45.74	
ECOG PS	0	140	104	74.29	36	25.71	0.456	105	75.00	35	25.00	0.443	63	45.00	77	55.00	0.014
	1	49	39	79.59	10	20.41		34	69.39	15	30.61		32	65.31	17	34.69	
p-Stage	I	92	72	78.26	20	21.74	0.417	66	71.74	26	28.26	0.584	45	48.91	47	51.09	0.24
	II	54	41	75.93	13	24.07		42	77.78	12	22.22		25	46.30	29	53.70	
	III	43	30	69.77	13	30.23		31	72.09	12	27.91		25	58.14	18	41.86	
Pleural invasion	Yes	14	13	92.86	1	7.14	0.217	10	71.43	4	28.57	0.898	6	42.86	8	57.14	0.565
	No	175	130	74.29	45	25.71		129	73.71	46	26.29		89	50.86	86	49.14	
Vascular invasion	Yes	9	6	66.67	3	33.33	0.805	6	66.67	3	33.33	0.927	6	66.67	3	33.33	0.505
	No	180	137	76.11	43	23.89		133	73.89	47	26.11		89	49.44	91	50.56	
Differentiation	High	10	7	70.00	3	30.00	0.960	7	70.00	3	30.00	0.915	4	40.00	6	60.00	0.732
	Intermediate	124	93	75.00	31	25.00		92	74.19	32	25.81		63	50.81	61	49.19	
	Low	55	43	78.18	12	21.82		40	72.73	15	27.27		28	50.91	27	49.09	

*ECOG PS* Eastern Cooperative Oncology Group performance score, *p* pathological, *PD-L1* programmed death ligand 1, *TIL* tumor-infiltrating lymphocyte, *TMB* tumor mutation burden

The mean TMB was 10.77 mutations per megabase (mut/Mb) and median TMB was 9.43 mut/Mb (Additional file 1: Figure S1A), which was similar to the results of TCGA (mean TMB, 8.1 mut/Mb; median TMB, 8.4 mut/Mb), and CHOICE (mean TMB, 11.8 mut/Mb) study [30, 31]. The common cutoffs for TMB include dichotomy, trichotomy, and quartering. In this study, we defined the high-TMB group as that with a TMB value at or above the median level and the low-TMB group as that with a TMB value below the median level. This definition for TMB cutoff is popular in the research setting because it is more helpful for us to clarify the relevant investigations due to its briefness. We observed that only ECOG PS = 0 was associated with a significantly higher rate of samples with low TMB (*P* = 0.014). There was no significant relationship between TMB and other clinical features (Table 1). Of note, patients with CNVs had significantly higher TMB than those without CNVs (*P* = 0.008; Additional file 1: Figure S1B). Two antibody assays (SP142 and E1L3N) were used to test PD-L1 expression. The representative IHC images were listed in Additional file 1: Figure S2A. A significant correlation of PD-L1 expression score between two assays was found (*R*^2^ = 0.782, *P* < 0.001; Additional file 1: Figure S2B) while E1L3N had a higher mean score (Additional file 1: Figure S2C). Using cutoff of 5% (E1L3N), PD-L1^+^ was observed in 24.3% of all cases (Additional file 1: Figure S4A), which was lower than it in Caucasian with LUSC [32]. Interestingly, in CHOICE study (includes both Chinese LUSC and LUAD), PD-L1 positivity rate was 23.1% using *H*-score ≥ 50, or 63.9% using > 1% tumor cell positive as a cutoff, which is consistent with those in the literature on the Western population [31]. Only young age was associated with significantly higher rate of PD-L1^+^ (31.43% VS. 15.48%, *P* = 0.011; Table 1). No significant association was observed between PD-L1 expression and other clinical parameters. Patients with CNVs had similar PD-L1 expression score to those without CNVs (*P* = 0.816; Additional file 1: Figure S1C). CD8^+^ TIL^+^ was observed in 78.8% of all samples (Additional file 1: Figure S4B) and representative IHC images of CD8^+^ TIL were showed in Additional file 1: Figure S3. There was no significant correlation between CD8^+^ TIL density and clinicopathological features. Patients with CNVs had similar CD8^+^ TIL density to those without CNVs (*P* = 0.317; Additional file 1: Figure S1D).

Although previous studies reported that there was no correlation between TMB and PD-L1 expression in NSCLC [14, 32], we explored these relationships not only between TMB and PD-L1 expression but also between TMB and CD8^+^ TIL density. Consistently, no correlation was found between TMB and PD-L1 expression (Spearman *R* = 0.052, *P* = 0.475; Additional file 1: Figure S4C), TMB and CD8^+^ TIL density (Spearman *R* = 0.026, *P* = 0.724; Additional file 1: Figure S4D). A significant positive correlation was found between PD-L1 expression and CD8^+^ TIL density (Spearman *R* = 0.232, *P* = 0.001; Additional file 1: Figure S4E).

We then investigated the associations between frequent genomic alterations and TMB, PD-L1 expression, or CD8^+^ TIL density. As shown in Fig. 1, *PIK3CA* amplification was associated with markedly higher TMB (*P* = 0.036; Fig. 1e) and *NFE2L2* mutation was associated with marginally higher TMB than those without (*P* = 0.069; Fig. 1h). There was no significant association between other frequent somatic mutations and TMB (Fig. 1c–d, f–g, i–p) or PD-L1 expression (Fig. 1q, s). *KEAP1* mutation was associated with dramatically lower CD8^+^ TIL density (*P* = 0.005; Fig. 1r) while other frequent somatic mutations had no impact on CD8+ TIL density (Figure 1R and 1 T). Using the online database, we further surveyed the impact of these frequent genomic alterations on six immune infiltrates. Consistent with our IHC results, we observed that only *KEAP1* mutation had the negative impact on CD8^+^ T cell abundance (*P* < 0.05; Additional file 1: Figure S5D). Both *TP53* and *KEAP1* mutations were independently associated with significantly lower DCs and neutrophil infiltrations (*P* < 0.05, *P* < 0.05, respectively; Additional file 1: Figures S5A and S5D). *PIK3CA* mutation was associated with significantly lower macrophage infiltration (*P* < 0.05; Additional file 1: Figure S5F) whereas other somatic mutations had no impact on the six immune infiltrates (Additional file 1: Figure S5). *SOX2* amplification was associated with significantly lower CD8^+^ T cell abundance (*P* < 0.01; Additional file 1: Figure S6F). Additionally, most of frequent CNVs including *FGFR1*, *EGFR*, and *PIK3CA* amplifications and loss of *CDKN2A* were associated with significantly lower six immune infiltrates (Additional file 1: Figure S6).

### Impact of TMB, PD-L1 expression, and CD8^+^ TIL density on DFS and OS

**Fig. 2:**
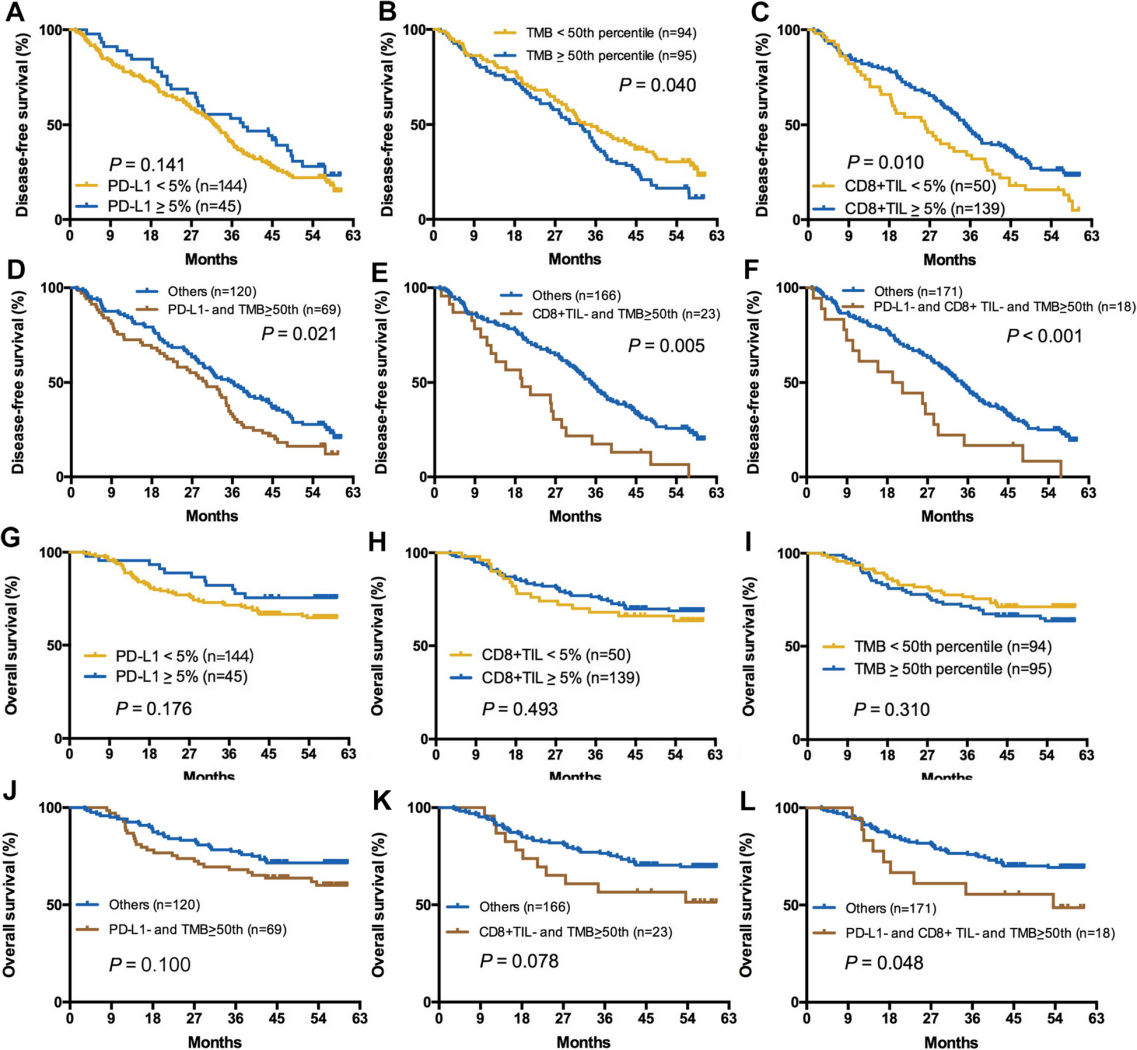
Impact of TMB, PD-L1, CD8+ TIL expression on DFS and OS. **a**–**c** Impact of TMB, PD-L1, CD8+ TIL expression on DFS. **d**–**f** Impact of TMB plus PD-L1 expression (**d**), TMB plus CD8+ TIL expression (**e**) and TMB plus PD-L1 plus CD8+ TIL expression (**f**) on DFS. **g–i** Impact of TMB, PD-L1, CD8+ TIL expression on OS. **j**–**l** Impact of TMB plus PD-L1 expression (**j**), TMB plus CD8+ TIL expression (**k**) and TMB plus PD-L1 plus CD8+ TIL expression (**l**) on OS

As shown in Fig. 2, high CD8^+^ TIL density and lower TMB were independently associated with significantly longer DFS (*P* = 0.010, *P* = 0.040, respectively). However, patients with different PD-L1 expression, CD8^+^ TIL density, and TMB had comparable OS (*P* = 0.176, *P* = 0.493, *P* = 0.310, respectively; Fig. 2g–i). Interestingly, a higher TMB cutoff was associated with a reduced statistical *P* value of both DFS and OS (Additional file 1: Figures S7 and S8). Although we chose 50% as the cutoff of PD-L1^+^ and CD8^+^ TIL^+^, there was no significant difference of DFS and OS (Additional file 1: Figures S7 and S8). The combination of TMB and PD-L1 or CD8^+^ TIL could discriminate the populations with different DFS (Fig. 2d, e) but numerically distinct OS (Fig. 2). When we combined TMB with PD-L1 and CD8^+^ TIL, the discriminatory power was significantly improved (Fig. 2f–l). Similarly, the discriminatory power was also improved along with the increase cutoffs of TMB (details in Additional file 1: Figures S7 and S8). Notably, the combination of TMB and CD8^+^ TIL density had a better discriminatory power than the combination of TMB and PD-L1 expression in stratifying patients with discrepant DFS and OS.

Univariate analysis indicated that stages II and III were significantly associated with shorter DFS (HR = 1.425, *P* = 0.033) and OS (HR = 2.952, *P* < 0.001) (Table 2). CD8^+^ TIL^−^, high TMB, PD-L1^−^ plus high TMB, and CD8^+^ TIL^−^ plus high TMB were independently associated with shorter DFS (*P* = 0.010, *P* = 0.040, *P* = 0.021, *P* = 0.005, respectively) while CD8^+^ TIL^+^ plus lower TMB was associated with longer DFS (*P* = 0.011). The combination of high TMB and PD-L1^−^ or CD8^+^ TIL^−^ did not reach the statistical significance in stratifying patients with different OS (*P* = 0.100, *P* = 0.078, respectively). Multivariate analysis revealed that stages II and III were associated with both markedly poor DFS (HR = 1.614, *P* = 0.005) and OS (HR = 2.844, *P* < 0.001). Only the combination of CD8^+^ TIL^+^ and lower TMB was associated with significantly longer DFS (HR = 0.506, *P* = 0.043) (Table 2).

**Table 2:** Univariate and multivariate analyses of clinical parameters on disease-free survival and overall survival

Factor	Univariate analysis			Multivariate analysis		
	HR (log rank)	95% CI	*P* value	HR (log rank)	95% CI	*P* value
Disease-free survival						
Sex (female/male)	0.818	0.416–1.606	0.559			
Age (≥ 65/< 65)	1.030	0.742–1.429	0.862			
Smoking (yes/no)	0.892	0.632–1.257	0.513			
PS (≥ 1/0)	1.092	0.759–1.571	0.636			
Stage (II–III/I)	1.425	1.028–1.973	0.033	1.614	1.156–2.255	0.005
Pleural invasion (yes/no)	1.629	0.900–2.950	0.107			
Vascular invasion (yes/no)	1.674	0.819–3.420	0.158			
Differentiation (high/non-high)	1.080	0.505–2.309	0.842			
PD-L1 (positive/negative)	0.702	0.474–1.041	0.141			
CD8^+^ TIL (positive/negative)	0.632	0.444–0.898	0.010	0.711	0.429–1.180	0.187
TMB (> median/≤ median)	1.454	1.047–2.019	0.040	1.122	0.652–1.929	0.677
PD-L1 negative and TMB > median VS. others	1.533	1.097–2.140	0.021	1.176	0.708–1.954	0.530
PD-L1 negative and TMB ≤ median VS. others	0.884	0.633–1.235	0.470			
PD-L1 positive and TMB ≤ median VS. others	0.506	0.272–0.938	0.031	0.506	0.261–0.979	0.043
PD-L1 positive and TMB > median VS. others	0.972	0.611–1.544	0.903			
CD8^+^ TIL negative and TMB > median VS. others	2.224	1.405–3.520	0.005	1.416	0.695–2.888	0.338
CD8^+^ TIL negative and TMB ≤ median VS. others	1.102	0.705–1.720	0.670			
CD8^+^ TIL positive and TMB ≤ median VS. others^#^	0.629	0.441–0.898	0.011			
CD8^+^ TIL positive and TMB > median VS. others	1.070	0.767–1.495	0.689			
Overall survival						
Sex (female/male)	1.012	0.317–3.229	0.984			
Age (≥ 65/< 65)	1.252	0.758–2.068	0.381			
Smoking (yes/no)	1.038	0.604–1.785	0.892			
PS (≤ 1/0)	0.995	0.562–1.761	0.987			
Stage (II–III/I)	2.952	1.685–5.172	< 0.001	2.844	1.606–5.038	< 0.001
Pleural invasion (yes/no)	1.425	0.613–3.309	0.411			
Vascular invasion (yes/no)	2.214	0.886–5.534	0.089	1.660	0.646–4.268	0.293
Differentiation (high/non-high)	1.892	0.758–4.727	0.172			
PD-L1 (positive/negative)	0.701	0.373–1.318	0.270			
CD8^+^ TIL (positive/negative)	0.825	0.476–1.430	0.493			
TMB (> median/≤ median)	1.366	0.824–2.263	0.227			
PD-L1 negative and TMB > median VS. others	1.591	0.960–2.638	0.100	1.425	0.822–2.468	0.207
PD-L1 negative and TMB ≤ median VS. others	0.818	0.485–1.380	0.452			
PD-L1 positive and TMB ≤ median VS. others	0.707	0.283–1.765	0.457			
PD-L1 positive and TMB > median VS. others	0.753	0.343–1.656	0.481			
CD8^+^ TIL negative and TMB > median VS. others	1.785	0.929–3.341	0.078	1.652	0.811–3.364	0.166
CD8^+^ TIL negative and TMB ≤ median VS. others	0.753	0.343–1.654	0.480			
CD8^+^ TIL positive and TMB ≤ median VS. others	0.819	0.480–1.398	0.464			
CD8^+^ TIL positive and TMB > median VS. others	1.031	0.614–1.730	0.909			

*HR* hazard ratio, *CI* confidence interval, *PS* performance score, *TMB* tumor mutation burden

^#^Reduction of degrees of freedom due to constants or linear dependent variables

### Classification of tumor immune microenvironment based on PD-L1 expression and CD8^+^ TIL density

**Fig. 3:**
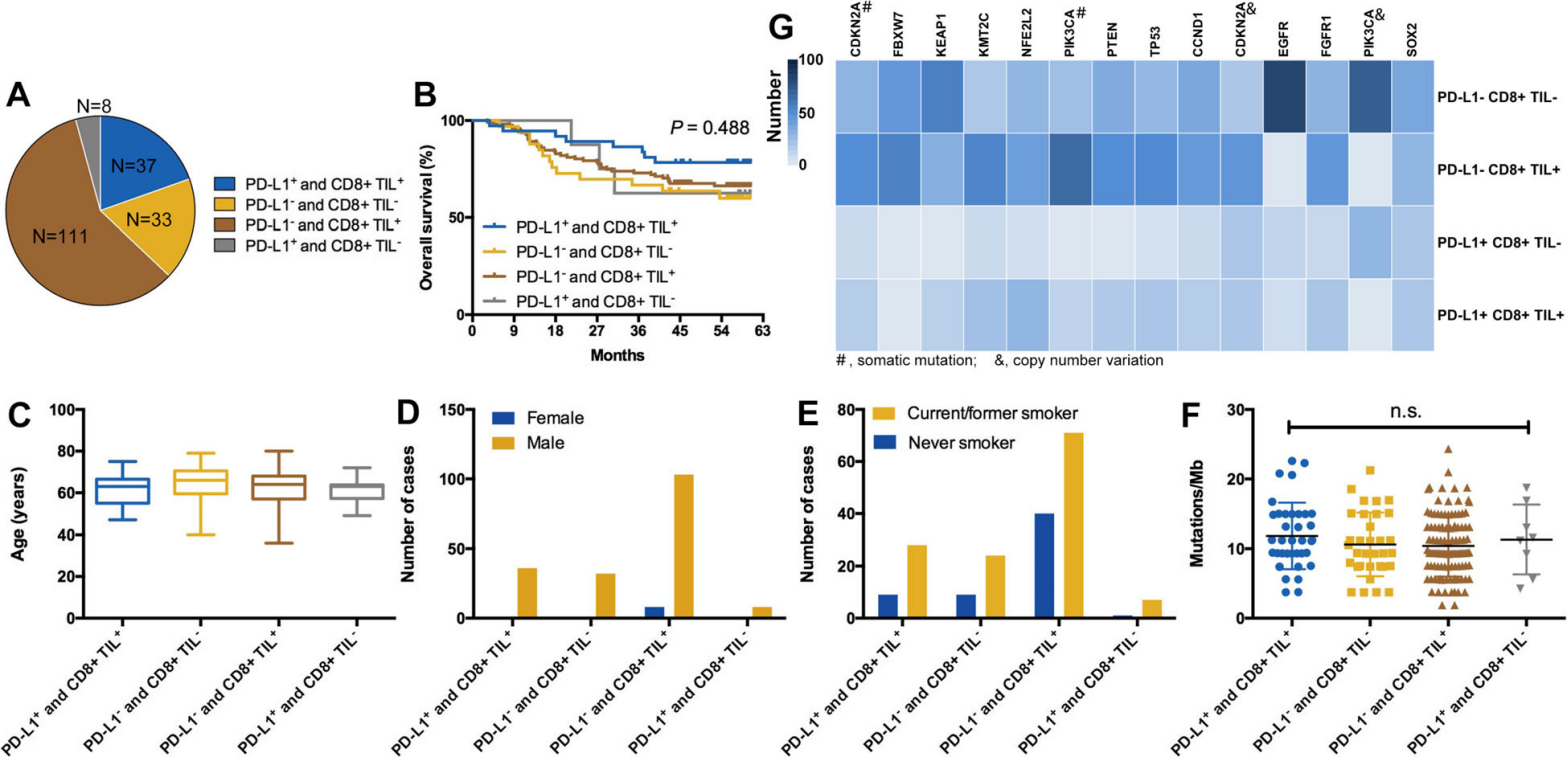
Classification of tumor immune microenvironment (TIME) based on PD-L1 and CD8+ TIL expression. **a** The distribution of distinct types of TIME. **b** Different types of TIME had similar prognosis. **c**–**f** Associations between types of TIME and age, sex, smoking history, and TMB. **g** Different genetic alterations among four types of TIME

According to previous classification method [33], we categorized the classification of tumor immune microenvironment (TIME) into four types based on PD-L1 expression and CD8^+^ TIL density (type I, PD-L1^+^CD8^+^ TIL^+^; type II, PD-L1^−^CD8^+^ TIL^−^; type III, PD-L1^+^CD8^+^ TIL^−^; type IV, PD-L1^−^CD8^+^ TIL^+^). We found that type IV (*n* = 111, 58.7%) TIME was dominant while type III (*n* = 8, 4.2%) was the least common in Chinese early-stage LUSC (Fig. 3a). The percentage of types I and II were 19.6% (*n* = 37) and 17.5% (*n* = 33), respectively (Fig. 3a). This classification had no prognostic value (*P* = 0.488; Fig. 3b). No significant correlation was found between types of TIME and age, sex, and smoking history (Fig. 3c–e). TMB was also comparable in different types of TIME (*P* > 0.05; Fig. 3f). Notably, we observed that types II and IV TIME had a significantly higher frequency of genetic alterations than types I and III (Fig. 3g).

### Subgroup analysis according to smoking status

**Fig. 4:**
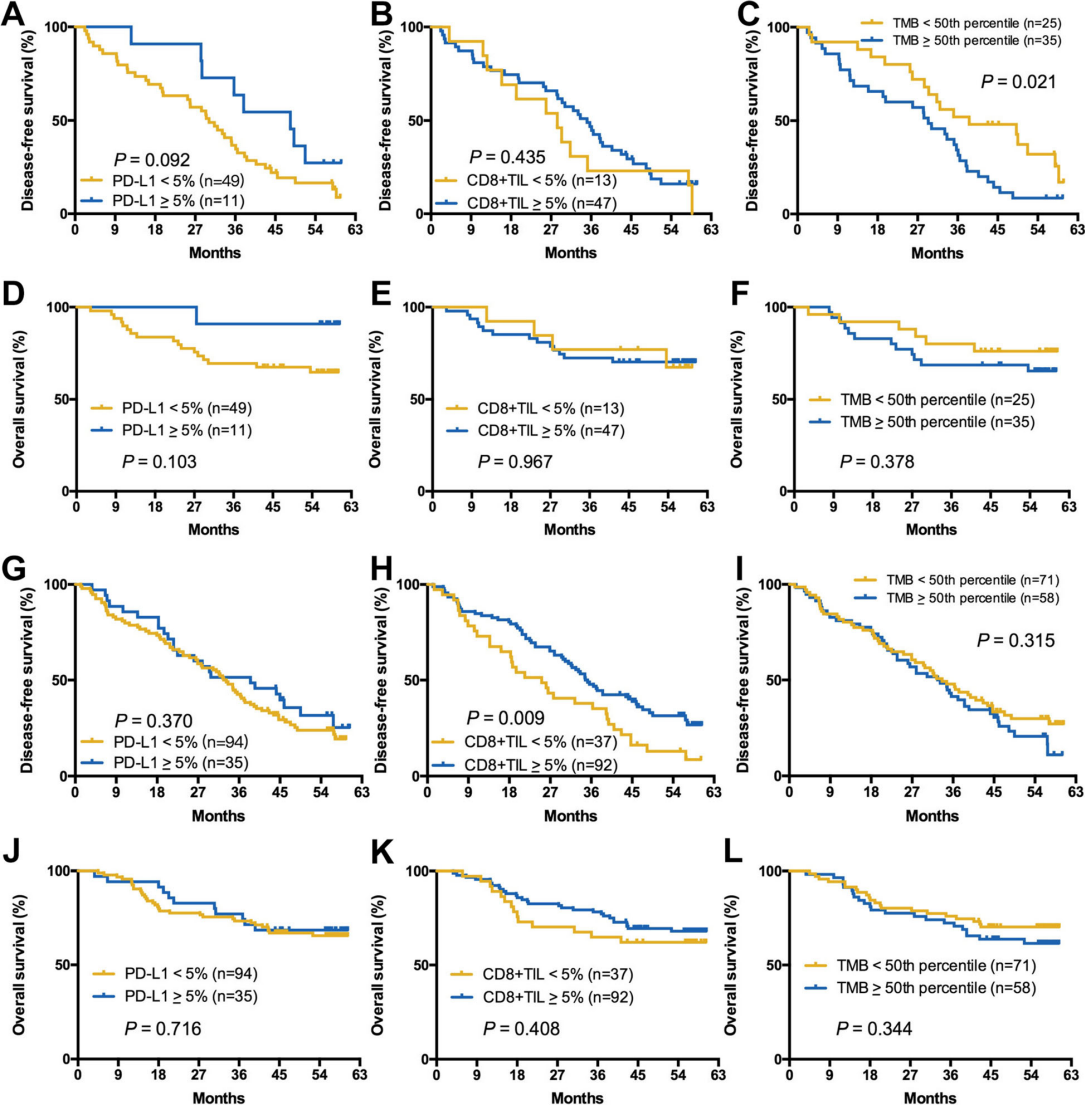
Subgroup analysis according to smoking status. **a**–**f** Impact of TMB, PD-L1, CD8+ TIL expression on DFS and OS in never-smoking group. **g**–**l** Impact of TMB, PD-L1, CD8+ TIL expression on DFS and OS in ever/current smoking group

Considering the impact of smoking history on TMB and TIME, and a high rate of never-smoker (31.7%) in this cohort, we further conducted the subgroup analysis of the associations between TMB, PD-L1 expression, CD8^+^ TIL density, and clinical features and outcomes according to the smoking status. As shown in Additional file 1: Table S2, the correlation between young age and PD-L1^+^ was only found in never-smoker (*P* = 0.048). There was no any correlation between TMB, PD-L1 expression, or CD8^+^ TIL density and other clinical parameters in both groups. In never-smoking group, PD-L1^+^ was associated with marginally significantly longer DFS (*P* = 0.092; Fig. 4a) but similar OS (*P* = 0.103; Fig. 4d). CD8^+^ TIL^+^ was not associated with both DFS and OS (Fig. 4b, e). High TMB was correlated with significantly longer DFS (*P* = 0.021; Fig. 4c) but not OS (*P* = 0.378; Fig. 4f). Intriguingly, in those with former/current smoking, both PD-L1 expression and TMB level were not correlated with DFS and OS (Fig. 4g, i, j, and l) while CD8^+^ TIL^+^ was associated with markedly longer DFS (*P* = 0.009; Fig. 4h).

## Discussion

Whole-exome sequencing of our cohort identified significant somatic mutations and CNVs that was consistent with previous publications on the genomic profile of early-stage LUSC [30, 31, 34, 35]. However, *SOX2* amplification was only found in 6.0% of all cases, which was significantly lower than other studies [30, 34]. Importantly, we identified a high frequency of *EML4-ALK* fusion (3.2%), one of the most common driver alterations in LUAD, which might mainly due to a high rate of never-smoker in our cohort, and infer the necessity to detect the common driver gene alterations in never-smokers with LUSC.

LUSC was previously found to have higher TMB than other solid tumors due to the close correlation to tobacco exposure [31, 36, 37]. However, the present study did not observe the association between smoking history and TMB level. In line with our results, Tatsuro et al. also did not find a correlation between the amount of smoking and TMB [35]. Furthermore, although the current cohort involved a large proportion of patients with never smoking, the mean and median TMB was similar to the results of TCGA and CHOICE study [30, 31]. These findings suggested that the relationship between tobacco exposure and TMB still remains further investigation. Intriguingly, patients with CNVs had significantly higher TMB than those without. This were reminiscent of an elegant study that examined the data from 5255 tumor/normal samples representing 12 tumor types from TCGA and found a positive correlation between somatic CNVs level and the total number of mutations [38], suggesting the potential value of CNVs for predicting the TMB level and its application for predicting who are most likely to benefit from immunotherapy. Of note, some kinds of CNVs, such as *FGFR1*, *EGFR*, and *PIK3CA* amplifications and loss of *CDKN2A*, were associated with significantly lower six immune infiltrates. This finding could partly explain that the fraction of copy number altered genome was highest in NSCLC patients treated with anti-PD-1/PD-L1 therapy but lack of durable benefit due to the importance of these immune infiltrates in antitumor immune response [14]. It also indicated that distinct kinds of CNVs would have a different effect on the immune response.

Understanding the interplay between molecular underpinnings and immune landscape may help improve strategies for precise immunotherapy [14, 39]. Towards this aim, we investigated the associations between frequent genomic alterations and PD-L1 expression, TMB, or CD8^+^ TIL density. The results showed that only *KEAP1* mutation was significantly associated with lower CD8^+^ TIL density, and *NFE2L2* mutation was associated with marginally higher TMB. Moreover, a recent study reported that there was a higher rate of *KEAP1/NFE2L2* >mutations in Chinese LUSC than those in Western populations [31]. KEAP1-NFE2L2 interaction plays a significant role in the dysregulation of oxidative stress pathway in lung cancer [40]. Genetic alterations of *KEAP1* or *NFE2L2* would destroy this process and lead to oncogenesis and drug and radio resistance in different types of solid tumors. Considering these findings, we could infer that tumor with *KEAP1* or *NFE2L2* mutation would have a higher level of oxidative stress, which could lead to the destruction of immune cells including CD8^+^ TILs and increased DNA damage level, resulting in the increase of somatic mutations of tumor cells. We also used the online database to calculate the abundance of tumor-infiltrating immune cells [28, 29]. Consistent with our IHC results, we observed that *KEAP1* mutation had the negative effect on CD8^+^ T cell abundance. *KEAP1* mutation was reported to be associated with poor response to adjuvant chemotherapy in both LUSC and LUAD [19, 34, 41]. Whether the relationship between *KEAP1* mutation and lower CD8^+^ T cell infiltration could explain the negative predictive value on adjuvant chemotherapy warrants further examinations.

Currently, TMB and PD-L1 expression are the two developed predictive biomarkers for anti-PD-1/PD-L1 therapy. We found no association between TMB and PD-L1 expression, which was consistent with previous findings. Yu et al. reported that PD-L1 protein expression was not correlated with TMB in both tumor cells and tumor-infiltrating immune cells of early-stage LUSC [32]. Rizvi et al. also found that TMB did not correlate with PD-L1 expression in patients with NSCLC treated with anti-PD-1/PD-L1 therapy [14]. Moreover, our study indicated that TMB did not correlate with CD8^+^ TIL density but a significant positive correlation was found between CD8^+^ TIL density and PD-L1 expression. Similar to previous reports [42, 43], positive CD8^+^ TIL density and lower TMB were independently associated with significantly longer DFS even though none of TMB, PD-L1 expression, or CD8^+^ TIL density were associated with OS. The combination of TMB and PD-L1 expression or CD8^+^ TIL density showed an improved yield in stratifying patients with discrepant DFS and OS, suggesting that the incorporation of these biomarkers into multivariable predictive and prognostic models worth further investigation in the future.

Furthermore, we classified the TIME into four types based on PD-L1 expression and CD8^+^ TIL density. Our data indicated that type IV TIME was dominant while type III was the least common in Chinese early-stage LUSC, which is in contrast to the previous studies [21, 44]. The potential reasons may include the different populations, testing methods and platforms, together with the discrepancy between the online RNA-seq and IHC staining data. As we know, type I TIME is most likely to benefit from anti-PD-1/PD-L1 monotherapy for the reason that these tumors have evidence of pre-existing intratumor T cells that are silenced by PD-L1 engagement. Constantly, the proportion of type I TIME in our cohort was similar to the response rate of patients with LUSC received single-agent anti-PD-1/PD-L1 checkpoint blockade [7–9]. We also found that different types of TIME had distinct genetic alterations but similar TMB, clinical variables, and prognosis, suggesting the unique shaping function of genomic landscape on immune phenotypes.

Additionally, we observed that high TMB was correlated with significantly longer DFS in never-smoker but not associated with DFS in former/current smoker. CD8^+^ TIL^+^ was not associated with DFS in never-smoker but associated with markedly longer DFS in former/current smoker. Both TMB level and CD8^+^ TIL+ were not associated with OS. PD-L1^+^ was not correlated with both DFS and OS in two groups. NSCLC of never-smokers are entirely different from these of former/current smokers. In never-smoking group, high TMB in tumors with never-smoking would mainly come from the intrinsic mechanism, which means the high possibility to be recognized and eliminated by the immune system. In this process, more and more CD8^+^ TILs would be induced and translated into the exhausted phenotype. Hence, high TMB was correlated with significantly longer DFS but CD8^+^ TIL+ was not associated with DFS in never-smoker. Conversely, high TMB in smokers caused by tobacco exposure could not predict the clinical outcome. However, CD8^+^ TILs in smokers could recognize and eliminate the malignant cells after exposure to the different carcinogens. Hence, high CD8^+^ TIL density was found to associate with markedly longer DFS in the current study. We must mention that the relatively small sample size and multiple different subsets based on distinct cutoffs of TMB, PD-L1 expression, CD8^+^ TIL density, and/or smoking status for these exploratory analyses would also be the potential reason for these discrepancies. Future investigations with a larger number of patients with LUSC are still needed.

## Conclusions

In summary, this large-scale study found *PIK3CA* amplification was associated with higher TMB but lower CD8^+^ T cells density while the common genetic alterations had no impact on PD-L1 expression. Combination of TMB and PD-L1/CD8+ TIL could be helpful to stratify the whole population into distinct DFS and OS subgroups. TIME classifications according to PD-L1 expression and CD8^+^ TIL density showed distinct genomic alterations but similar TMB, clinical variables, and prognosis. Notably, TMB and CD8^+^ TIL+ might have a distinct role in patients with different smoking status. These findings shed a light and might be helpful to guide future design of personalized immunotherapy trials in LUSC.

## Additional file

Additional file 1: **Figure S1.** (A) the distribution of TMB; (B–D) the comparison of TMB (B), PD-L1 expression (C), and CD8^+^ TIL density (D) between patients with CNVs and those without CNVs. **Figure S2.** (A) The representative immunohistochemical images of PD-L1 expression, (B) the correlations of PD-L1 expression score between SP142 and E1L3N, and (C) the distribution of PD-L1 expression score in each antibody assay. **Figure S3.** The representative immunohistochemical images of CD8+ TIL expression. **Figure S4.** The distribution of different PD-L1 expression (A) and CD8^+^ TIL density (B); (C) correlation between TMB and PD-L1 expression, (F) TMB and CD8^+^ TIL density, and (E) PD-L1 expression and CD8^+^ TIL density. **Figure S5.** Association between frequent somatic mutations and six immune cells infiltration. **Figure S6.** Association between frequent CNVs and six immune cells infiltration. **Figure S7.** DFS at PD-L1 expression cutoff of 5% (A) and 50% (B); CD8^+^ TIL expression cutoff of 5% (C) and 50% (D); TMB cutoff of 25th (E), 50th (F), 75th (G) and 90th (H) percentile; TMB plus PD-L1 at TMB cutoff of 25th (I), 50th (J), 75th (K) and 90th (L) percentile; TMB plus CD8^+^ TIL expression at TMB cutoff of 25th (M), 50th (N), 75th (O) and 90th (P) percentile. **Figure S8.** OS at PD-L1 cutoff of 5% (A) and 50% (B); CD8^+^ TIL expression cutoff of 5% (C) and 50% (D); TMB cutoff of 25th (E), 50th (F), 75th (G) and 90th (H) percentile; TMB plus PD-L1 at TMB cutoff of 25th (I), 50th (J), 75th (K) and 90th (L) percentile; TMB plus CD8^+^ TIL expression at TMB cutoff of 25th (M), 50th (N), 75th (O) and 90th (P) percentile. **Table S1.** The relationship between EGFR amplification/EML4-ALK fusion and clinicopathological features. **Table S2.** Baseline characteristics of included patients according to smoking status. (DOCX 3849 kb)

## Data Availability

Not applicable.

## Abbreviations

CI: Confidence interval
CNV: Copy number variation
DC: Dendritic cell
DFS: Disease-free survival
ECOG PS: Eastern Cooperative Oncology Group performance status
FFPE: Formalin-fixed and paraffin-embedded
HR: Hazard ratios
IHC: Immunohistochemistry
LUAD: Lung adenocarcinoma
LUSC: Lung squamous cell carcinoma
NSCLC: Non-small-cell lung cancer
OS: Overall survival
PD-1: Programmed cell death 1
PD-L1: Programmed cell death ligand 1
SNV: Single nucleotide variant
TIL: Tumor-infiltrating lymphocytes
TIME: Tumor immune microenvironment
TMB: Tumor mutation burden
TPS: Tumor proportion score
