# Supplementary material for: Genomic landscape and its correlations with tumor mutational burden, PD-L1 expression, and immune cells infiltration in Chinese lung squamous cell carcinoma
Source: J Hematol Oncol. 2019 Jul 12;12:75. doi: 10.1186/s13045-019-0762-1 (PMC6625041; doi:10.1186/s13045-019-0762-1)
Supplement: Supplementary file 1 — Figure S1. (A) the distribution of TMB; (B–D) the comparison of TMB (B), PD-L1 expression (C), and CD8+ TIL density (D) between patients with CNVs and those without CNVs. Figure S2. (A) The representative immunohistochemical images of PD-L1 expression, (B) the correlations of PD-L1 expression score between SP142 and E1L3N, and (C) the distribution of PD-L1 expression score in each antibody assay. Figure S3. The representative immunohistochemical images of CD8+ TIL expression. Figure S4. The distribution of different PD-L1 expression (A) and CD8+ TIL density (B); (C) correlation between TMB and PD-L1 expression, (F) TMB and CD8+ TIL density, and (E) PD-L1 expression and CD8+ TIL density. Figure S5. Association between frequent somatic mutations and six immune cells infiltration. Figure S6. Association between frequent CNVs and six immune cells infiltration. Figure S7. DFS at PD-L1 expression cutoff of 5% (A) and 50% (B); CD8+ TIL expression cutoff of 5% (C) and 50% (D); TMB cutoff of 25th (E), 50th (F), 75th (G) and 90th (H) percentile; TMB plus PD-L1 at TMB cutoff of 25th (I), 50th (J), 75th (K) and 90th (L) percentile; TMB plus CD8+ TIL expression at TMB cutoff of 25th (M), 50th (N), 75th (O) and 90th (P) percentile. Figure S8. OS at PD-L1 cutoff of 5% (A) and 50% (B); CD8+ TIL expression cutoff of 5% (C) and 50% (D); TMB cutoff of 25th (E), 50th (F), 75th (G) and 90th (H) percentile; TMB plus PD-L1 at TMB cutoff of 25th (I), 50th (J), 75th (K) and 90th (L) percentile; TMB plus CD8+ TIL expression at TMB cutoff of 25th (M), 50th (N), 75th (O) and 90th (P) percentile. Table S1. The relationship between EGFR amplification/EML4-ALK fusion and clinicopathological features. Table S2. Baseline characteristics of included patients according to smoking status. (DOCX 3849 kb) [file 13045_2019_762_MOESM1_ESM.docx]

**Supplemental Material**

**Genomic landscape and its correlations with TMB, PD-L1 expression and immune cells infiltration in lung squamous cell carcinoma**

Tao Jiang, Chunyan Wu, Jinpeng Shi, Zhengwei Dong, Likun Hou, Chao Zhao, Xuefei Li, Beibei Mao, Wei Zhu, Xianchao Guo, Henghui Zhang, Ji He, Xiaoxia Chen, Chunxia Su, Shengxiang Ren, Caicun Zhou

**Supplemental Methods....................................................................................Page 2.**

**Supplemental Figure S1..................................................................................Page 4.**

**Supplemental Figure S2..................................................................................Page 5**

**Supplemental Figure S3..................................................................................Page 6.**

**Supplemental Figure S4..................................................................................Page 7.**

**Supplemental Figure S5..................................................................................Page 8.**

**Supplemental Figure S6..................................................................................Page 9.**

**Supplemental Figure S7..................................................................................Page 10.**

**Supplemental Figure S8..................................................................................Page 12. Supplemental Table S1....................................................................................Page 14.**

**Supplemental Table S2....................................................................................Page 15.**

**Supplemental Methods**

**DNA Extraction and Library Construction**

Genomic DNA was extracted from all included samples. The matched peripheral blood leukocytes were utilized as the source for germline DNA control. DNA was extracted from the tumor tissues using Qiagen DNeasy FFPE DNA kit (Qiagen, Hilden, Germany) according to the manufacturer’s instructions. Following quality check was conducted using Agilent 2100 Bioanalyzer (Life Technologies, USA) per manufacturer’s recommended protocol. Genomic DNA was sheared into 150-200 base pairs (bp) fragments with Covaris M220 Focused-ultrasonicator TM Instrument (Covaris, Massachusetts, USA). Fragmented DNAs were constructed via KAPA Hyper Prep Kit (Illumina platforms) (KAPA Biosystems, Massachusetts, USA) according to the manufacturer’s protocol. Multiple indexing adaptors were ligated to the ends of the DNA fragments to prepare them for hybridization onto a flow cell. Purification and size selection of the library were performed using AMPure XP magnetic beads (Beckman Coulter, Brea, CA, USA). The concentration and quality of the library was determined by using the Qubit 3.0 system (Invitrogen) and Bioanalyzer 2100 (Agilent, Agilent HS DNA Reagent, 5067-4627).

**Whole-exome sequencing**

DNA libraries were subjected to whole-exome capture with xGen Exome Research Panel v1.0 (Integrated DNA Technologies), which spans a 39 Mb target region (19,396 genes) of the human genome and covers 51 Mb of end-to-end tiled space. Human Cot-1 DNA (Life Technologies) and xGen universal blocking oligos (Integrated DNA Technologies) were added as blocking reagents to reduce non-specific hybridization. The capture reaction was performed with NimbleGen SeqCap EZ Hybridization and Wash Kit (Roche) and Dynabeads M-270 (Life Technologies) according to manufacturers’ protocols. The captured samples were sequenced on an Illumina HiSeq X-TEN platform with a paired-end run of 2 × 150bp. The quality of each read was initially verified using the software embedded in the HiSeq X-TEN sequence. The sequencing depth was more than 150×. A FASTQ file was generated for each tested sample for sequence alignment and converted to a BAM file for further analysis (All FASTQ files are available on request).

**Data filtering and variant calling**

The generated sequencing reads were initially parsed with FLEXBAR for adapter trimming and low quality bases were filtered out[1]. Raw sequence reads were mapped to the human reference genome (hg19) by Burrows-Wheeler Aligner (BWA) aligner v0.7.12[2]. Duplicated reads were then removed from the aligned and sorted BAM files by using Picard 2.2.1. GATK v3.8 was utilized to do local realignment around potential small insertions and deletions (Indels) and base recalibration for next step mutation calling procedures. We used MuTect v1.1.7 to detect single nucleotide variants (SNVs) and Strelka v1.0.14 to call small Indels[3, 4]. Tumor-normal paired sample calling was processed during the mutation calling procedure, in order to filter out individual’s private germline mutations. The resulted somatic mutations above variant allele frequency (VAF) of 2% were selected for annotation. ANNOVAR was run to screen the nonsynonymous mutations in the exonic region for further study. The candidate variants with a minor allele frequency above 0.2% as recorded in either the population database EXAC (The Exome Aggregation Consortium) v.03 or the Genome Aggregation Database (gnomAD) were filtered out for further identification of the somatic mutations.

**References**

1. Dodt M, Roehr JT, Ahmed R, Dieterich C. FLEXBAR-Flexible Barcode and Adapter Processing for Next-Generation Sequencing Platforms. Biology (Basel) 2012; 1: 895-905.

2. Li H, Durbin R. Fast and accurate short read alignment with Burrows-Wheeler transform. Bioinformatics 2009; 25: 1754-1760.

3. Cibulskis K, Lawrence MS, Carter SL et al. Sensitive detection of somatic point mutations in impure and heterogeneous cancer samples. Nat Biotechnol 2013; 31: 213-219.

4. Saunders CT, Wong WS, Swamy S et al. Strelka: accurate somatic small-variant calling from sequenced tumor-normal sample pairs. Bioinformatics 2012; 28: 1811-1817.

**
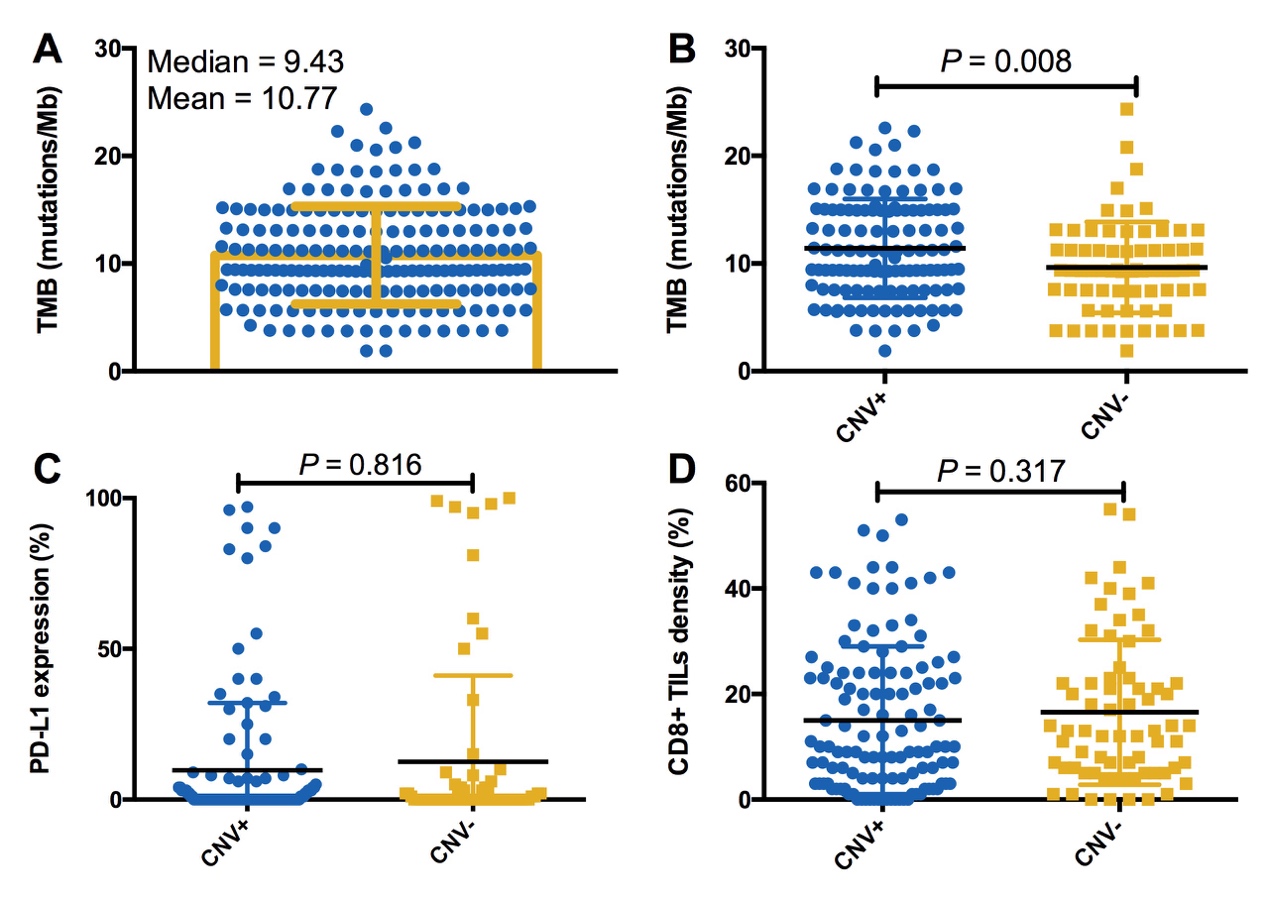
**

**Supplemental Figure S1. A). the distribution of TMB in all samples; B-D). the comparison of TMB (B), PD-L1 expression (C), and CD8+ TIL density (D) between patients with copy number variations (CNVs) and those without CNVs.**

**
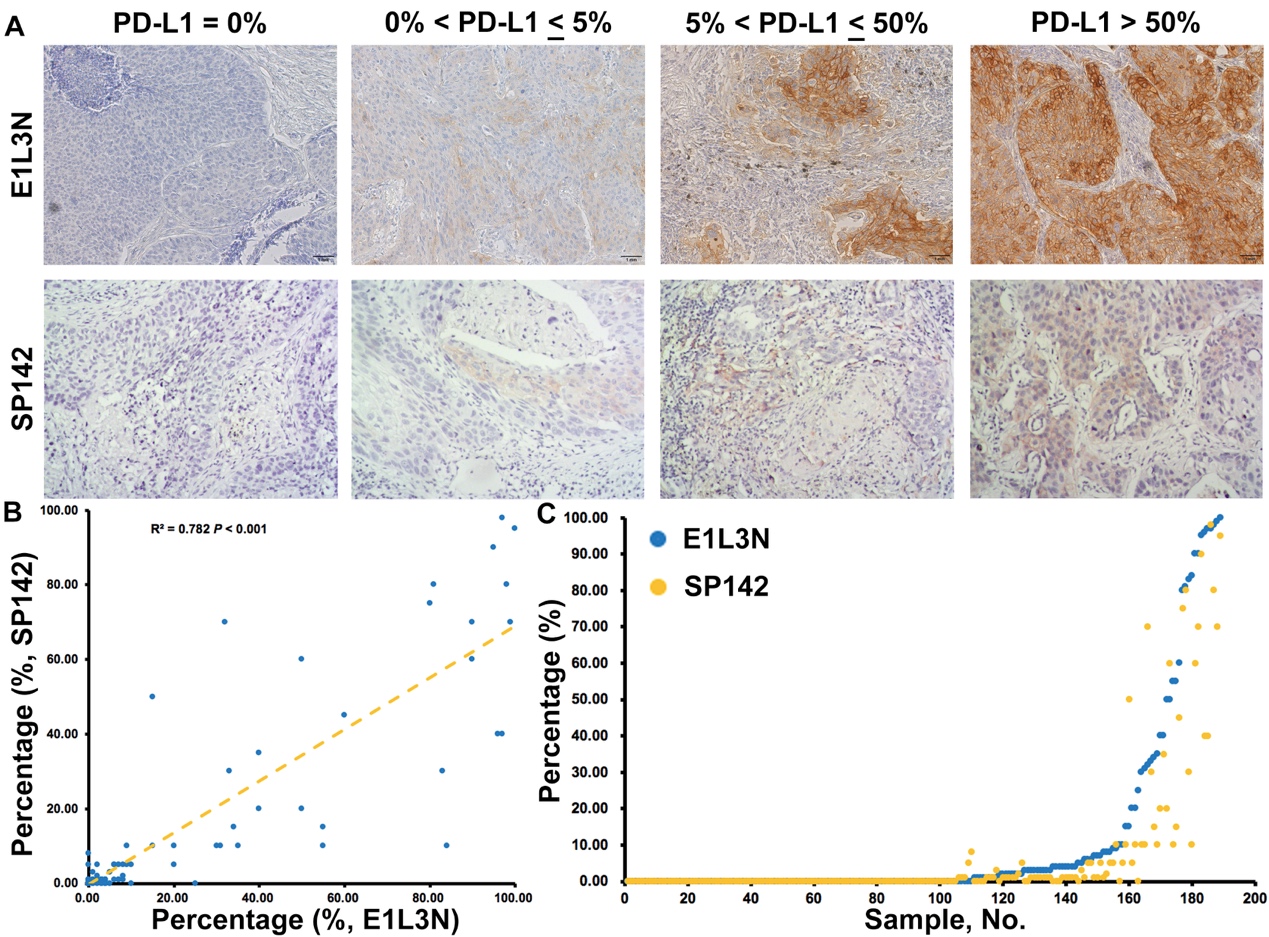
**

**Supplemental Figure S2. Detection of PD-L1 expression. A). the representative immunohistochemical images of PD-L1 expression by using two antibody assays; B). the correlations of PD-L1 expression score between SP142 and E1L3N; C). the distribution of PD-L1 expression score in each antibody assay.**

**
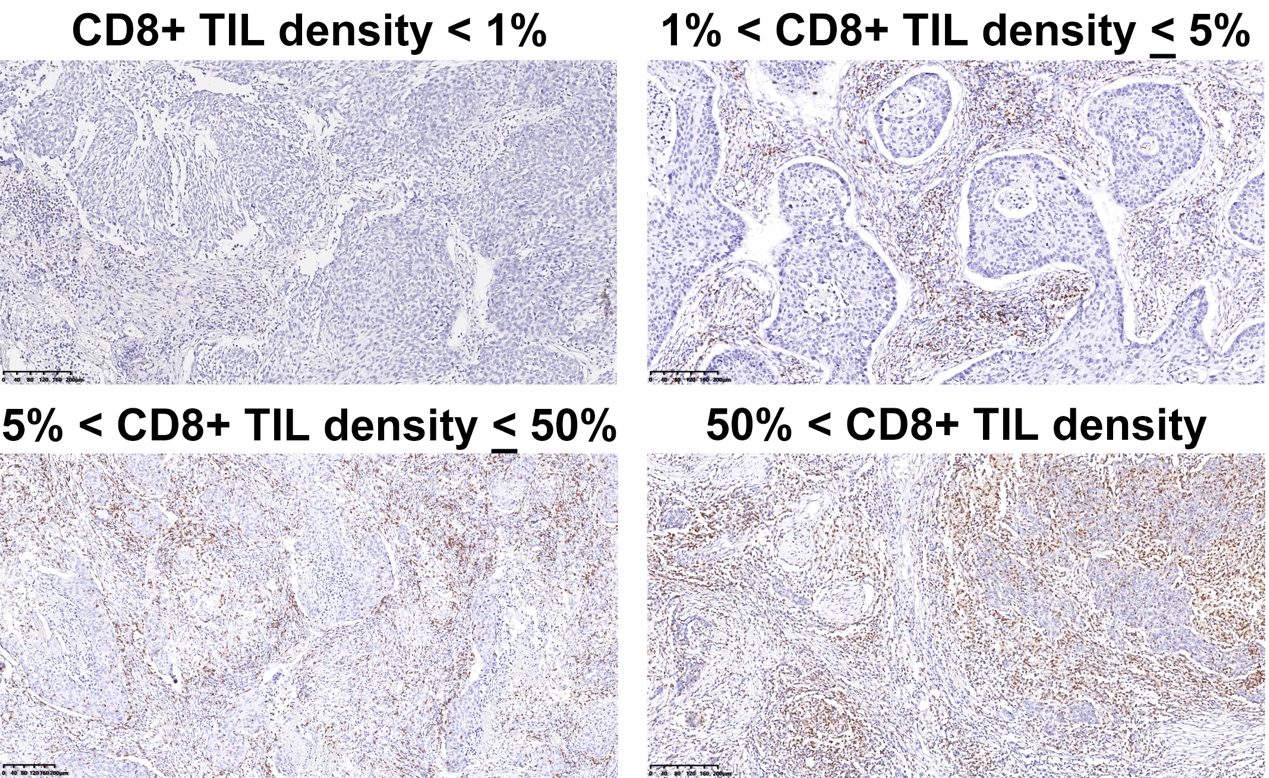
**

**Supplemental Figure S3. The representative immunohistochemical images of CD8+ TIL expression.**

**
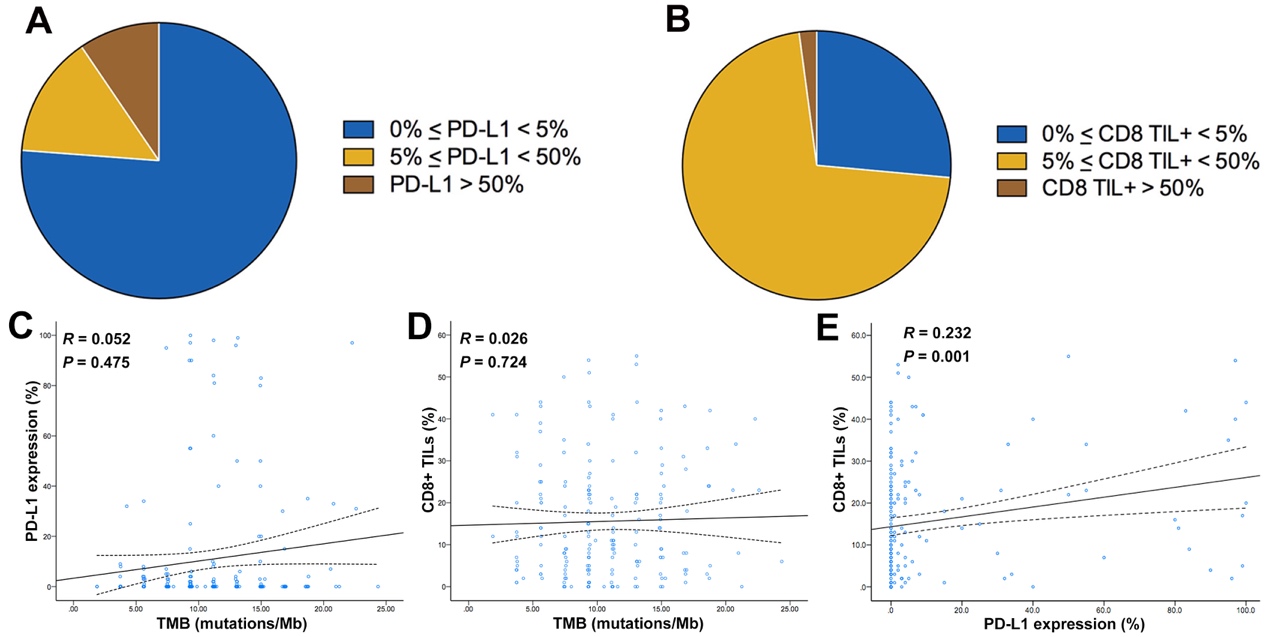
**

**Supplemental Figure S4. The distribution of PD-L1 and CD8+ TIL expression, and their correlations with TMB. (A). The distribution of different PD-L1 expression level; (B). The distribution of different CD8+ TIL density; (C). Correlation between TMB and PD-L1 expression; (F). Correlation between TMB and CD8+ TIL density; (E). Correlation between PD-L1 expression and CD8+ TIL density.**

**
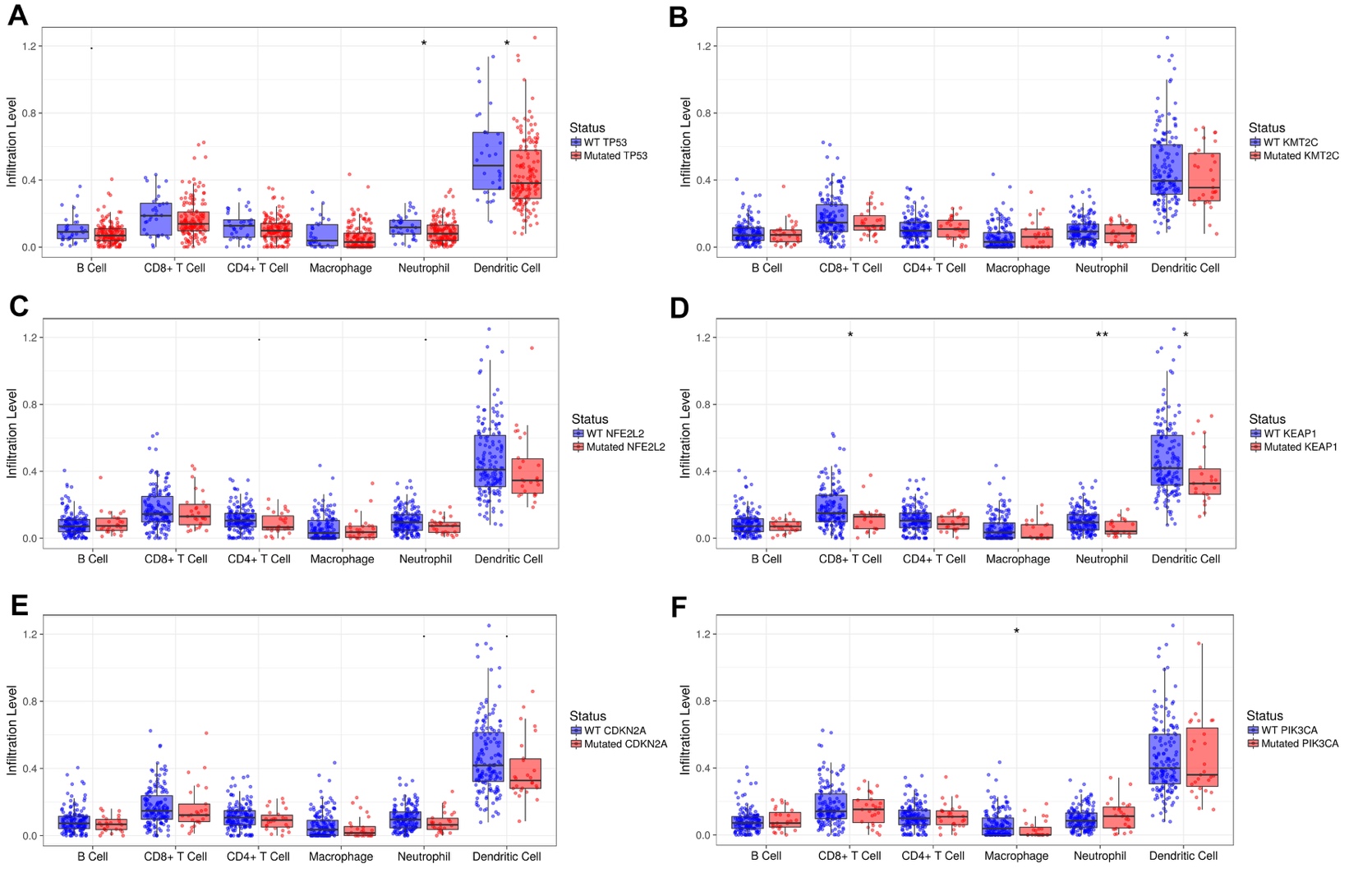
**

**Supplemental Figure S5. Association between frequent somatic mutations including *TP53* (A), *KMT2C* (B), *KEAP1* (C), *CDKN2A* (D), *NFE2L2* (E), *PIK3CA* (F) mutation and six immune cells infiltration via using online database.**

**
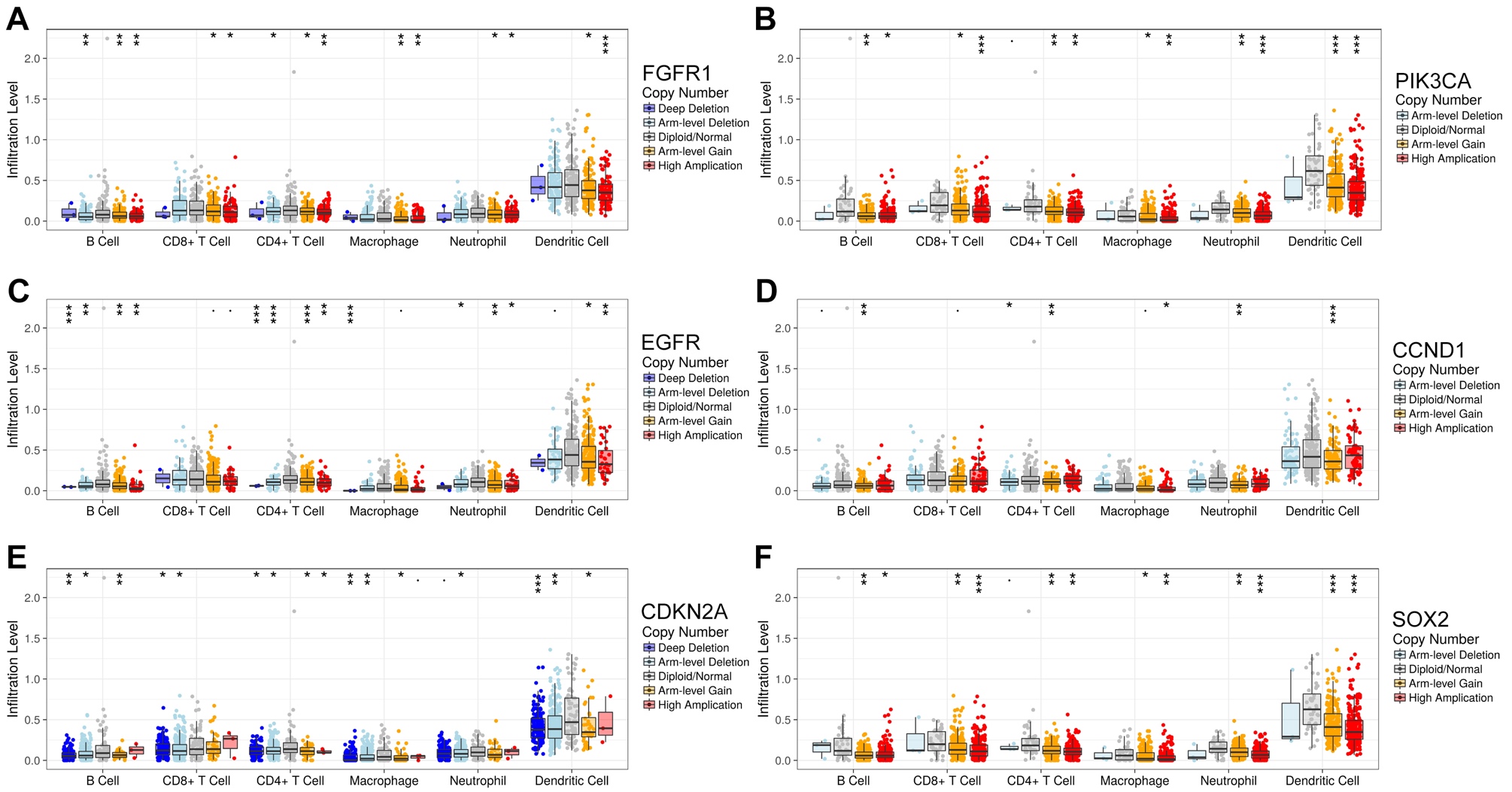
**

**Supplemental Figure S5. Association between frequent CNVs including *FGFR1* (A), *PIK3CA* (B), *EGFR* (C), *CCND1* (D) and *SOX2* (E) amplification, and loss of *CDKN2A* (F) and six immune cells infiltration via using online database.**

**
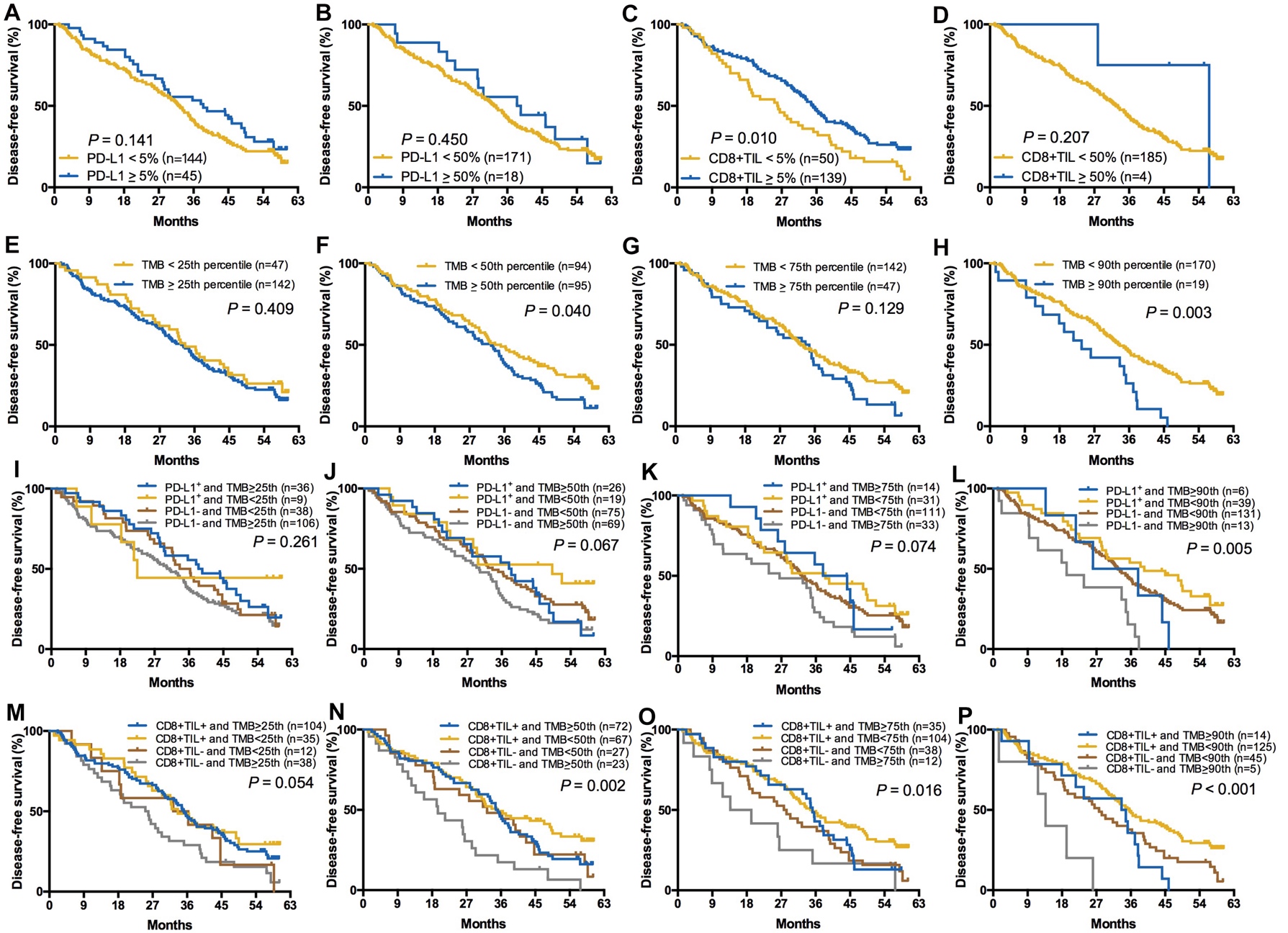
**

**Supplemental Figure S7. Association between disease-free survival (DFS) and TMB level, PD-L1 and CD8+ TIL expression alone or in combination. No significant difference of DFS was observed at PD-L1 expression cutoff of 5% (A) or cutoff of 50% (B); A significant difference of DFS was observed at CD8+ TIL expression cutoff of 5% (C) but not 50% (D); A significant difference of OS was observed at TMB cutoff of 50^th^ (F) and 90^th^ percentile (H) but not 25^th^ (E) and 75^th^ (G) percentile; Combination of TMB and PD-L1 cannot stratify the populations with different prognosis at TMB cutoff of 25^th^ (I), 50^th^ (J) or 75^th^ (K) percentile; Combination of TMB and PD-L1 can stratify the populations into two groups with different prognosis at TMB cutoff of 90^th^ (L) percentile; Combination of TMB and CD8+ TIL expression cannot stratify the populations with different prognosis at TMB cutoff of 25^th^ (M); Combination of TMB and CD8+ TIL expression can stratify the populations into two groups with different prognosis at TMB cutoff of 50^th^ (N), 75^th^ (O) or 90^th^ (P) percentile.**

**
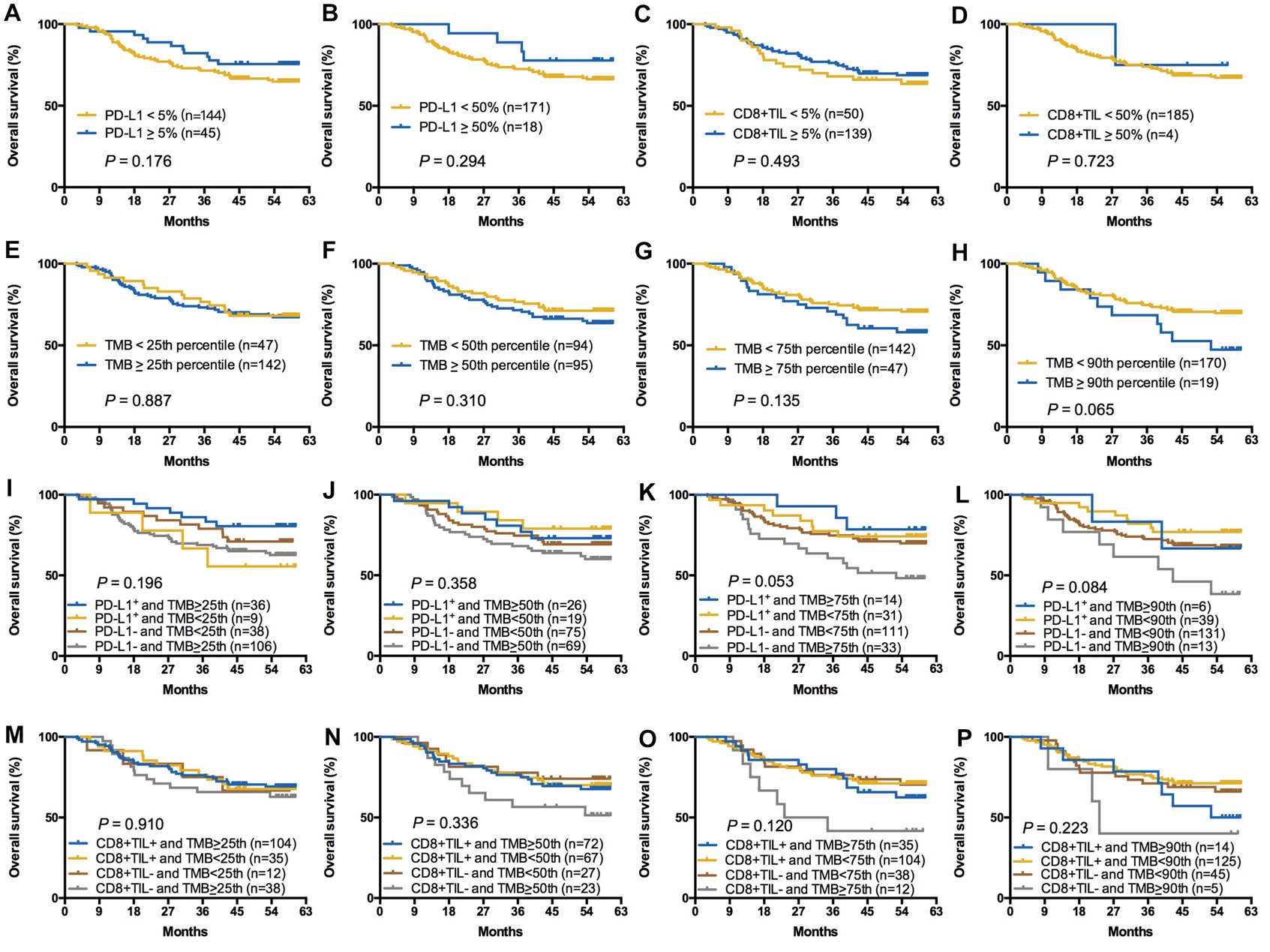
**

**Supplemental Figure S8. Prognostic value of TMB, PD-L1 and CD8+ TIL expression alone or in combination. (A-B). No significant difference of overall survival (OS) was observed at PD-L1 expression cutoff of 5% (A) or cutoff of 50% (B); (C-D). No significant difference of OS was observed at CD8+ TIL expression cutoff of 5% (C) or cutoff of 50% (D); (E-G). No significant difference of OS was observed at TMB cutoff of 25th (E), 50th (F) or 75th (G) percentile; (H). A marginally statistical significant P value was observed at TMB cutoff of 90th percentile; (I-J). Combination of TMB and PD-L1 cannot stratify the populations with different prognosis at TMB cutoff of 25th (I) or 50th (J) percentile; (K-L). Combination of TMB and PD-L1 can stratify the populations into two groups with different prognosis at TMB cutoff of 75th (K) or 90th (L) percentile; (M-N). Combination of TMB and CD8+ TIL expression cannot stratify the populations with different prognosis at TMB cutoff of 25th (M) or 50th (N) percentile; (O-P). Combination of TMB and CD8+ TIL expression can stratify the populations into two groups with different prognosis at TMB cutoff of 75th (O) or 90th (P) percentile.**

| **Supplemental Table S1. The relationship between EGFR amplification/EML4-ALK fusion and clinicopathological features.** | | | | | | | | | | | | | | | | | | | | | | |  |  |
| --- | --- | --- | --- | --- | --- | --- | --- | --- | --- | --- | --- | --- | --- | --- | --- | --- | --- | --- | --- | --- | --- | --- | --- | --- |
|  | |  | | **Total** | | **EGFR amplification** | | | | | | |  | **EML4-ALK fusion** | | | | | |  | | |  |  |
|  | |  | |  |  | **Yes** | | | **No** | | | | ***P*** | **Yes** | | | **No** | | | ***P*** | | |  |  |
|  | |  | | **N** | | **N** | | **%** | **N** | | **%** | |  | **N** | | **%** | **N** | | **%** |  |  |  |  |  |
| **Age** | | <65 | | 105 | | 33 | | 31.43 | 72 | | 68.57 | | 0.917 | 2 | | 1.90 | 103 | | 98.10 | 0.487 | | |  |  |
|  | | >=65 | | 84 | | 27 | | 32.14 | 57 | | 67.86 | |  | 4 | | 4.76 | 80 | | 95.24 |  | | |  |  |
| **Sex** | | Male | | 179 | | 57 | | 31.84 | 122 | | 68.16 | | 0.820 | 6 | | 3.35 | 173 | | 96.65 | 0.735 | | |  |  |
|  | | Female | | 10 | | 3 | | 30.00 | 7 | | 70.00 | |  | 0 | | 0.00 | 10 | | 100.0 |  | | |  |  |
| **Smoking** | | Never | | 60 | | 17 | | 28.33 | 43 | | 71.67 | | 0.492 | 4 | | 6.67 | 58 | | 96.67 | 0.073 | | |  |  |
|  | | Current/former | | 129 | | 43 | | 33.33 | 86 | | 66.67 | |  | 2 | | 1.55 | 125 | | 96.90 |  | | |  |  |
| **ECOG PS** | | 0 | | 140 | | 44 | | 31.43 | 96 | | 68.57 | | 0.874 | 5 | | 3.57 | 135 | | 96.43 | 0.958 | | |  |  |
|  | | 1 | | 49 | | 16 | | 32.65 | 33 | | 67.35 | |  | 1 | | 2.04 | 48 | | 97.96 |  | | |  |  |
| **p-Stage** | | I | | 92 | | 23 | | 25.00 | 69 | | 75.00 | | 0.052 | 2 | | 2.17 | 90 | | 97.83 | 0.727 | | |  |  |
|  | | II | | 54 | | 23 | | 42.59 | 31 | | 57.41 | |  | 3 | | 5.56 | 51 | | 94.44 |  | | |  |  |
|  | | III | | 43 | | 14 | | 32.56 | 29 | | 67.44 | |  | 1 | | 2.33 | 42 | | 97.67 |  | | |  |  |
| **Pleural invasion** | | Yes | | 14 | | 4 | | 28.57 | 10 | | 71.43 | | 0.974 | 2 | | 14.29 | 12 | | 85.71 | 0.095 | | |  |  |
|  |  | No | | 175 | | 56 | | 32.00 | 119 | | 68.00 | |  | 4 | | 2.29 | 171 | | 97.71 |  | | |  |  |
| **Vascular invasion** | | Yes | | 9 | | 3 | | 33.33 | 6 | | 66.67 | | 0.793 | 1 | | 11.11 | 8 | | 88.89 | 0.676 | | |  |  |
|  |  | No | | 180 | | 57 | | 31.67 | 123 | | 68.33 | |  | 5 | | 2.78 | 175 | | 97.22 |  | | |  |  |
| **Differentiation** | | High | | 10 | | 1 | | 10.00 | 9 | | 90.00 | | 0.242 | 0 | | 0.00 | 10 | | 100.0 | 0.735 | | |  |  |
|  |  | Intermediate | | 124 | | 38 | | 30.65 | 86 | | 69.35 | |  | 4 | | 3.23 | 120 | | 96.77 |  | | |  |  |
|  | | Low | | 55 | | 21 | | 38.18 | 34 | | 61.82 | |  | 2 | | 3.64 | 53 | | 96.36 |  | | |  |  |
| ECOG PS, Eastern Cooperative Oncology Group performance score; p, pathological; PD-L1, programmed death ligand 1; TIL, tumor infiltrating lymphocyte. | | | | | | | | | | | | | | | | | | | | | | |  |  |
| **Supplemental Table S2. Baseline characteristics of included patients according to smoking status.** | | | | | | | | | | | | | | | | | | | | | | |  |  |
|  |  | | **Total** | | **PD-L1-** | | **PD-L1+** | | | ***P* value** | | **CD8+ TIL high** | | | **CD8+ TIL low** | | | ***P* value** | | | **TMB high** | **TMB low** | | ***P* value** |
|  |  | | **N** | | **N** | | **N** | | |  |  | **N** | | | **N** | | |  |  |  | **N** | **N** | |  |
| ***Never-smoker*** |  | |  | |  | |  | | |  | |  | | |  | | |  | | |  |  | |  |
| **Age** | <65 | | 36 | | 26 | | 10 | | | 0.048 | | 29 | | | 7 | | | 0.609 | | | 19 | 17 | | 0.285 |
|  | >=65 | | 24 | | 23 | | 1 | | |  | | 18 | | | 6 | | |  | | | 16 | 8 | |  |
| **Sex** | Male | | 50 | | 40 | | 10 | | | 0.765 | | 39 | | | 11 | | | 0.779 | | | 29 | 21 | | 0.815 |
|  | Female | | 10 | | 9 | | 1 | | |  | | 8 | | | 2 | | |  | | | 6 | 4 | |  |
| **ECOG PS** | 0 | | 49 | | 40 | | 9 | | | 0.677 | | 37 | | | 12 | | | 0.474 | | | 32 | 17 | | 0.048 |
|  | 1 | | 11 | | 9 | | 2 | | |  | | 10 | | | 1 | | |  | | | 3 | 8 | |  |
| **p-Stage** | I | | 30 | | 27 | | 3 | | | 0.299 | | 24 | | | 6 | | | 0.754 | | | 21 | 9 | | 0.067 |
|  | II | | 21 | | 16 | | 5 | | |  | | 17 | | | 4 | | |  | | | 9 | 12 | |  |
|  | III | | 9 | | 7 | | 2 | | |  | | 6 | | | 3 | | |  | | | 5 | 4 | |  |
| **Pleural invasion** | Yes | | 4 | | 4 | | 0 | | | 0.755 | | 2 | | | 2 | | | 0.426 | | | 3 | 1 | | 0.861 |
|  | No | | 56 | | 45 | | 11 | | |  | | 45 | | | 11 | | |  | | | 32 | 24 | |  |
| **Vascular invasion** | Yes | | 2 | | 2 | | 0 | | | 0.804 | | 2 | | | 0 | | | 0.907 | | | 1 | 1 | | 0.627 |
|  | No | | 58 | | 47 | | 11 | | |  | | 45 | | | 13 | | |  | | | 34 | 24 | |  |
| **Differentiation** | High | | 4 | | 2 | | 2 | | | 0.305 | | 4 | | | 0 | | | 645.000 | | | 4 | 0 | | 0.221 |
|  | Intermediate | | 41 | | 33 | | 8 | | |  | | 30 | | | 11 | | |  | | | 22 | 19 | |  |
|  | Low | | 15 | | 14 | | 1 | | |  | | 13 | | | 2 | | |  | | | 9 | 6 | |  |
| ***Current/ever smoker*** | | |  | |  | |  | | |  | |  | | |  | | |  | | |  |  | |  |
| **Age** | <65 | | 69 | | 46 | | 23 | | | 0.089 | | 53 | | | 16 | | | 0.139 | | | 33 | 36 | | 0.483 |
|  | >=65 | | 60 | | 48 | | 12 | | |  | | 39 | | | 21 | | |  | | | 25 | 35 | |  |
| **Sex** | Male | | 129 | | 94 | | 35 | | | - | | 92 | | | 37 | | | - | | | 48 | 71 | | - |
|  | Female | | 0 | | 0 | | 0 | | |  | | 0 | | | 0 | | |  | | | 0 | 0 | |  |
| **ECOG PS** | 0 | | 91 | | 64 | | 27 | | | 0.316 | | 68 | | | 23 | | | 0.185 | | | 45 | 46 | | 0.113 |
|  | 1 | | 38 | | 30 | | 8 | | |  | | 24 | | | 14 | | |  | | | 13 | 25 | |  |
| **p-Stage** | I | | 63 | | 46 | | 17 | | | 0.971 | | 43 | | | 20 | | | 0.452 | | | 26 | 37 | | 0.410 |
|  | II | | 33 | | 25 | | 8 | | |  | | 25 | | | 8 | | |  | | | 20 | 13 | |  |
|  | III | | 33 | | 23 | | 10 | | |  | | 24 | | | 9 | | |  | | | 12 | 21 | |  |
| **Pleural invasion** | Yes | | 10 | | 9 | | 1 | | | 0.369 | | 8 | | | 2 | | | 0.789 | | | 4 | 6 | | 0.998 |
|  | No | | 119 | | 85 | | 34 | | |  | | 84 | | | 35 | | |  | | | 54 | 65 | |  |
| **Vascular invasion** | Yes | | 7 | | 4 | | 3 | | | 0.600 | | 4 | | | 3 | | | 0.672 | | | 2 | 5 | | 0.613 |
|  | No | | 122 | | 90 | | 32 | | |  | | 88 | | | 34 | | |  | | | 56 | 66 | |  |
| **Differentiation** | High | | 6 | | 4 | | 2 | | | 0.904 | | 3 | | | 3 | | | 0.471 | | | 2 | 4 | | 0.868 |
|  | Intermediate | | 83 | | 60 | | 23 | | |  | | 62 | | | 21 | | |  | | | 38 | 45 | |  |
|  | Low | | 40 | | 30 | | 10 | | |  | | 27 | | | 13 | | |  | | | 18 | 22 | |  |
| ECOG PS, Eastern Cooperative Oncology Group performance score; p, pathological; PD-L1, programmed death ligand 1; TIL, tumor infiltrating lymphocyte. | | | | | | | | | | | | | | | | | | | | | | | | |
